# Short-term exposure to ambient temperature variability and myocardial infarction hospital admissions: A nationwide case-crossover study in Sweden

**DOI:** 10.1371/journal.pmed.1004607

**Published:** 2025-05-20

**Authors:** Wenli Ni, Massimo Stafoggia, Siqi Zhang, Petter Ljungman, Susanne Breitner, Jeroen de Bont, Tomas Jernberg, Dan Atar, Alexandra Schneider, Stefan Agewall

**Affiliations:** 1 Department of Clinical Sciences, Danderyd Hospital, Karolinska Institutet, Stockholm, Sweden; 2 Institute of Epidemiology, Helmholtz Zentrum München—German Research Center for Environmental Health (GmbH), Neuherberg, Germany; 3 Institute for Medical Information Processing, Biometry, and Epidemiology, Pettenkofer School of Public Health, LMU Munich, Munich, Germany; 4 Department of Epidemiology, Lazio Regional Health Service, ASL Roma 1, Rome, Italy; 5 Institute of Environmental Medicine, Karolinska Institutet, Stockholm, Sweden; 6 Department of Cardiology, Danderyd Hospital, Stockholm, Sweden; 7 Institute of Clinical Medicine, University of Oslo, Oslo, Norway; 8 Department of Cardiology, Oslo University Hospital Ulleval, Oslo, Norway; South African Medical Research Council, SOUTH AFRICA

## Abstract

**Background:**

Climate change threatens human health and general welfare via multiple dimensions. However, the associations of short-term exposure to temperature variability, a crucial aspect of climate change, with myocardial infarction (MI) hospital admissions remains unclear.

**Methods and findings:**

This population-based nationwide study employed a time-stratified, case-crossover design to investigate the association between ambient temperature variability and MI hospital admissions among 233,617 patients recorded in the SWEDEHEART registry in Sweden between 2005 and 2019. High-resolution (1 × 1 km) daily mean ambient temperature was assigned to patients’ residential areas. Temperature variability was calculated as the difference between the same-day (as the MI event) ambient temperature and the average temperature over the preceding 7 days. An upward temperature shift represents a rise in the current day’s temperature relative to the 7-day average, while a downward temperature shift indicates a corresponding decrease. A conditional logistic regression model with distributed lag non-linear model was applied to estimate the association between ambient temperature variability and total MI (encompassing all MI types), ST-segment elevation myocardial infarction (STEMI) and non-ST-segment elevation myocardial infarction (NSTEMI) hospital admissions at lag 0–6 days. Potential effect modifiers, such as sex, history of diseases, and season, were also examined.

The patients had an average age of 70.6 years, and 34.5% of them were female. Our study found that an upward temperature shift was associated with increased risks of total MI (encompassing all MI types), STEMI, and NSTEMI hospital admissions at lag 0 day, with odds ratios (OR, 95% confidence intervals [CIs]) of 1.009 (1.005, 1.013; *p* < 0.001), 1.014 (1.006, 1.022; *p* < 0.001), and 1.007 (1.001, 1.012; *p* = 0.014) per 1 °C increase, respectively. These associations attenuated and became non-significant over lags 1–6 days. Furthermore, a downward temperature shift was associated with increased risks of hospital admissions for total MI (encompassing all MI types) at a lag of 2 days with an OR (95% CI): 1.003 (1.001, 1.005; *p* = 0.014), and for STEMI at lags 2 and 3 days with ORs (95% CI): 1.006 (1.002, 1.010; *p* = 0.001) and 1.005 (1.001, 1.008; *p* = 0.011), per 1 °C decrease, respectively. Conversely, higher downward temperature shifts were associated with decreased risks of total MI (encompassing all MI types) and NSTEMI at lag 0 day. No significant associations were observed at other lag days for downward temperature shifts. Males and patients with diabetes had higher MI hospitalization risks from upward temperature shift exposure, while downward temperature shift exposure in cold seasons posed greater MI hospitalization risks.

A methodological limitation was the use of ambient temperature variability as a proxy for personal exposure, which, while practical for large-scale studies, may not precisely reflect individual temperature exposure.

**Conclusions:**

This nationwide study contributes insights that short-term exposures to higher temperature variability—greater upward or downward temperature shifts—are associated with an increased risk of MI hospitalization. Our finding highlights the cardiovascular health threats posed by higher temperature variability, which are anticipated to increase in frequency and intensity due to climate change.

## Author summary

**Why was this study done?:**

- Climate change has led to increasing air temperature changes, but its specific associations with heart attack (myocardial infarction) risk are not well understood.
- Very few studies have examined how different directions of air temperature changes (sudden warming versus cooling) affect heart attack risk.
- Understanding how air temperature changes affect heart attack risk could help improve public health strategies.

**What did the researchers do and find?:**

- We studied over 230,000 heart attack hospitalizations in Sweden between 2005 and 2019 to investigate how sudden air temperature changes affected their risk of hospital admissions.
- We found that upward temperature shift exposures were associated with an increased risk of hospital admissions for heart attacks on the same day, while downward temperature shift exposures were associated with increased risk 2–3 days later.
- Men and people with diabetes were more vulnerable to heart attacks hospital admissions during upward temperature shifts. The risk of hospital admissions due to heart attacks during temperature drops was higher in cold seasons than in warm seasons.

**What do these findings mean?:**

- These findings suggest that air temperature changes, upward and downward temperature shifts, are associated with increased heart attack hospital admissions.
- Healthcare providers could use this information to better prepare for increased heart attack cases during periods of significant temperature changes.
- Although our study looked at outdoor temperature changes, we could not measure exactly what temperatures each person was exposed to, which is a limitation of our findings.

## Abstract 3

Wenli Ni and colleagues use a time-stratified case-crossover design to examine the association between ambient temperature variability and hospital admissions for myocardial infarction among 233,617 patients in Sweden.

## Introduction

Climate change represents a critical global challenge, causing more frequent and intense extreme weather events [1]. In addition to rising average temperatures, recent decades have seen an increasing trend in global temperature variability [2]. Temperature variability refers to the degree to which temperature fluctuates over time. Temperature variability has been identified as a health determinant, including an increased risk of cardiovascular diseases [2–5], but its health associations are less well studied than those of non-optimal temperature. The health effects resulting from temperature variability, which can happen at any time of the year, could be similar to or more severe than those caused by exposure to extreme temperatures [6]. A global study revealed an annual global death toll of almost 1.8 million deaths associated with temperature variability, accounting for 3.4% of all deaths [2].

Myocardial infarction (MI), identified as one of the gravest presentations of coronary artery disease (CAD), carries the potential to precipitate sudden cardiac mortality [7]. This pathophysiological condition can be delineated into two principal categories: ST-segment elevation MI (STEMI) and non-ST-segment elevation MI (NSTEMI). Previous studies have suggested that non-optimal temperature exposures, encompassing cold spells and heat waves, are associated with an increased risk of MI occurrence and death [8–13]. However, the impact of temperature variability, a significant element of climate change, on MI remains unclear, with findings across studies demonstrating inconsistency [14–26]. A city-based study in Beijing, China, which uniquely explored directional temperature variability, found that daily mean temperature differences at the first percentile and 99th percentile were associated with increased MI hospitalizations [25].

Parsing directional temperature variability effects (differentiating the effects of upward versus downward temperature shift) enables a more nuanced elucidation of the potentially asymmetric impacts of different changing weather patterns on MI risk. Additionally, recognizing that weather instability is a continuous process and that human physiological and behavioral adaptation may occur over days [27], it would be beneficial to assess the temperature variability over extended time periods (e.g., over a week) that better reflect adaptive processes.

Therefore, this nationwide case-crossover study aimed to investigate the association between short-term exposure to temperature variability (upward and downward temperature shifts), calculated as the difference between the same-day and the average ambient temperature over the preceding 7 days, and the risk of MI hospitalization from 2005 to 2019 within a high-quality database in Sweden.

## Methods

### Study population

The study was conducted in Sweden, a Nordic country located in Northern Europe. According to the Köppen–Geiger climate classification, the majority of Sweden experiences a humid continental climate (Dfb), characterized by mild summers and cold winters in central and southern Sweden [28,29]. The subarctic climate (Dfc), with longer, colder winters and shorter, cooler summers, is prevalent in northern Sweden [28,29]. Sweden spans a range of latitudes, leading to climatic variation across regions. During the study period (2005–2019), the average mean temperature in our study was 3.50 °C in the northern regions, 6.58 °C in the central areas, and 7.74 °C in the southern regions. This south-to-north gradient shows that southern and central parts of Sweden had higher average temperatures, with differences of up to about 4 °C compared to northern areas. Over the study period, the annual average temperature in Sweden increased from 6.50 °C in 2005 to 7.50 °C in 2019 (S1 Fig).

Data were obtained from the SWEDEHEART, a nationwide population-based registry in Sweden that consecutively enrolls all patients hospitalized due to symptomatology indicative of acute coronary syndrome, as well as patients undergoing coronary angiography or cardiac surgery irrespective of indication [30]. The diagnosis of MI (International Classification of Diseases, 10th Revision [ICD-10]: I21) was established based on the clinical assessment conducted by attending physicians on patient admission. MI events were further stratified by infarction type: STEMI and NSTEMI. Furthermore, data on demographic and clinical parameters, including age, sex, body mass index (BMI), smoking status, socioeconomic status, historical medical conditions, and medication intake were collected. This population-based nationwide study included 233,617 patients who were recorded as being hospitalized for MI, consisting of 73,318 patients with STEMI, 159,679 patients with NSTEMI, and an additional 620 patients where the type of MI was not specified, spanning from January 2005 to December 2019 (S2 Fig for flowchart). These patients who were included in the study had complete temperature variability and area of residence data.

This study has been conducted as part of the European Union (EU) project EXHAUSTION [31], which prospectively defined analyses of MI events in relation to heat and cold exposures. The present investigation, focusing on temperature variability as a potential risk factor for MI, was not based on a prespecified analysis plan. Rather, the research question emerged during ongoing analyses through interdisciplinary discussions with clinical collaborators (including cardiologists), informed by emerging literature on temperature variability and physiological adaptation mechanisms. The study was approved by the Swedish Ethical Review Authority (2020-04252) in accordance with the ethical principles outlined in the Declaration of Helsinki. SWEDEHEART complies with Swedish law, where informed consent is waived for quality registry data used for quality improvement and health research. All patients are informed about their participation in the registry and have the right to opt out or have their data erased upon request.

### Exposure assessment

High-resolution daily mean ambient temperature data, covering the entirety of Sweden at a spatial resolution of 1 × 1 km, were derived through a three-stage approach based on hybrid spatiotemporal regression models [32]. This process utilized data from multiple sources, including satellite land surface temperature (LST) data, monitored observed temperature data, and spatiotemporal predictors related to land use and land cover. The LST data were obtained from the Terra Moderate Resolution Imaging Spectroradiometer (MODIS) instrument, specifically the MOD11A1 Version 6.1 product. This product, provided by NASA, offers daily per-pixel Land Surface Temperature and Emissivity (LST&E) at a spatial resolution of 1 km. Observed meteorological data for Sweden were sourced from the Swedish Meteorological and Hydrological Institute (SMHI). Initially, missing daily LST data were imputed via collocated estimates of ambient temperature from atmospheric models. Subsequently, annual calibration of monitored ambient temperature against imputed LSTs was conducted using additional predictors on land cover, elevation, and population density. In the final analysis, the model was utilized to forecast daily temperatures for all grid cells devoid of monitors. Ultimately, daily mean ambient temperatures were obtained for each 1-km squared cell across Sweden from 2005 to 2019, with no missing data. Cross-validation estimated model performance was excellent, with an R-squared (R^2^) of 0.94 [32]. Finally, patients who experienced MI with hospital admissions in the registry were assigned daily ambient temperature exposures based on their area of residence (using Swedish administrative areas, parish-level during 2005–2014 and district-level during 2015–2019) and hospital admission date.

Daily concentrations of particulate matter with a diameter less than 2.5 μm (PM_2.5_), nitrogen dioxide (NO_2_), and ozone (O_3_) were estimated within a spatial resolution of 1 × 1 km grid encompassing the entirety of Sweden. These estimations were generated through the utilization of a nationally implemented machine learning model, which has been described in detail elsewhere [33]. The model’s performance displayed negligible bias and the model was able to predict most of the variability, with cross-validated *R*^2^ in the range of 0.64–0.78 for out-of-bag samples and 0.37–0.61 for held-out monitors [33].

### Definition of temperature variability

**Fig 1:**
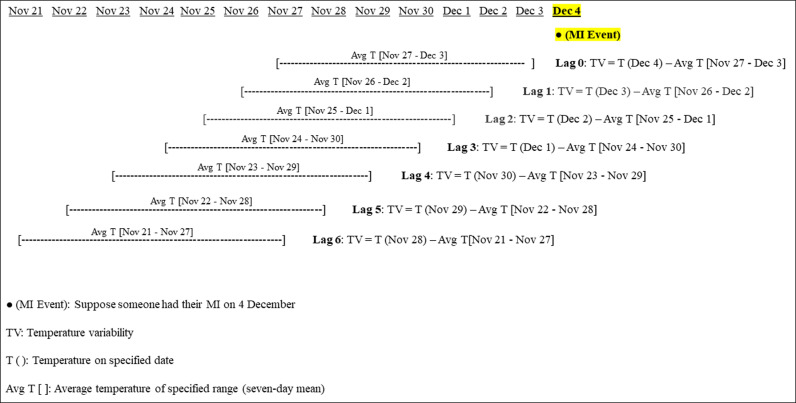
Temperature variability calculation for lags 0–6 for an example MI event on December 4. Note: MI, myocardial infarction.

Temperature variability was assessed by calculating the difference between the same-day (as the MI event) and the average temperature recorded over the preceding 7 days. This methodology is detailed in Fig 1, which illustrates the temperature variability calculations across lags 0–6 for an example MI event on December 4. A positive value indicates an increase compared to the 7-day mean, reflecting an upward swing in temperature (upward temperature shift). Conversely, a negative value indicates a decrease compared to the 7-day mean, indicating a downward swing in temperature (downward temperature shift). This approach captures fluctuations relative to the recent temperature profile and incorporates multiday trends, thereby better-reflecting timescales relevant to human acclimatization processes. Moreover, distinguishing the impacts of upward and downward temperature shift (temperature variability) can provide valuable insights into how climate-driven temperature fluctuations contribute to the risk of MI, yielding more interpretable indicators of the effects associated with both upward and downward temperature shifts and a more refined characterization of how shifting weather patterns may disproportionately impact MI in divergent directions.

### Statistical analysis

We applied a time-stratified, case-crossover design incorporating a conditional logistic regression model with distributed lag non-linear model (DLNM) to assess the association between short-term exposure to temperature variability and total MI hospitalization (encompassing all MI types) and its subtypes, STEMI and NSTEMI at lag 0–6 days. For each participant, the exposure experienced on the specific day of MI occurrence (referred to as the “case” day) was compared with exposures experienced on similar weekdays within the same month and year (referred to as “control” days). Each case was matched with 3–4 control days, depending on the month’s duration, consistently matching the day of the week (e.g., all Mondays in January 2016). We opted for calendar months rather than fixed 28-day periods to maintain consistency with common temporal patterns and to facilitate ease of interpretation. This methodology ensures that control days do not overlap with the 7-day period used for calculating temperature variability for the case day, as they are always separated by at least 7 days. This time-stratified, case-crossover design allows for robust control of potential confounders characterized by temporal stability over brief periods, e.g., demographic characteristics. By choosing control days within the same temporal strata, we could also adjust for confounding factors such as long-term trends, seasonal variation, and the day of the week [34,35]. Specifically, matching within the week accounted for potential confounding associated with weekly patterns. Similarly, matching within the month addressed potential seasonal confounding, and matching within the year controlled for confounding from long-term temporal trends. Given the acute nature of MI and the risk of overfitting, where extending the lag period beyond 6 days could result in the same day incorrectly serving as both hazard and control in time-stratified, case-crossover designs, we examined the association between short-term exposure to temperature variability and MI across a 0–6-day lag period.

In our preliminary analyses, we included temperature variability as a natural cubic spline with three degrees of freedom in a conditional logistic regression to investigate the exposure–response association (S3 Fig). By selecting three degrees of freedom, we aimed to adequately capture potential nonlinear relationships between temperature variability and MI hospitalizations without introducing overfitting, which can occur when a model is unnecessarily complex with too many degrees of freedom. We observed an approximate threshold of 0, with slightly divergent slopes below and above this value (S3 Fig). Consequently, to examine the associations of both the upward and downward temperature shift (directional temperature variability) with MI, we employed the threshold function within DLNM, setting 0 as the threshold value. Specifically, the “crossbasis” function was applied, consisting of a threshold (“thr”) function for the exposure–response relationship (upward and downward temperature shift–MI relationship), in addition to the lag-response dimension modeled using a natural cubic spline with two interior knots at evenly spaced log values of lag days, based on prior literature and our own experience [36,37]. The formula is defined in S1 Text. We examined single-day lag associations from lag 0–6 days, crucial for identifying acute risk exposure windows. This strategy also helps mitigate the potential bias introduced by emphasizing cumulative associations, which may overlap with the calculation of temperature variability itself.

Furthermore, we performed subgroup analyses to distinguish between the first and the recurrent MI. Additionally, regional analyses were conducted to explore the potential geographic differences by investigating Sweden’s southern, central, and northern regions.

We conducted comprehensive effect modification analyses by incorporating potential effect modifiers as interaction terms in the models to explore the interaction between temperature variability and various potential effect modifiers. To quantitatively assess if the association of temperature variability with MI hospitalizations differed significantly between subgroups, we employed a two-sided *z*-test. The test statistic was calculated using the formula provided in S2 Text. These effect modifiers encompassed a range of demographic and lifestyle factors, including age (<65 versus ≥65 years), sex (male versus female), smoking status (current or former versus never smoker), and education (low, indicating education up to high school level or less, versus high, indicating any post-high school education). Furthermore, we examined the influence of pre-existing conditions (yes versus no) such as diabetes, CAD (defined as a history of MI, percutaneous coronary intervention, or coronary artery bypass surgery), heart failure, hypertension, percutaneous coronary intervention, stroke, chronic obstructive pulmonary disease (COPD), and peripheral vascular disease. Additionally, we explored the impact of medication use (yes versus no), encompassing the any medications; any heart medications; any anti-hypertensive medications; specific subtypes of heart or anti-hypertensive medication such as angiotensin-converting enzyme (ACE) inhibitors, angiotensin II receptor blockers (A2 blockers), beta-blockers, digoxin, long-acting nitro, calcium channel blockers, diuretics, and aldosterone inhibitors; any anticoagulant medications and specific subtypes such as oral anticoagulants, DOAC (including apixaban, dabigatran etexilate, edoxaban, and rivaroxaban), aspirin, platelet inhibitors, and clopidogrel; anti-diabetic medications; as well any lipid-lowering medications, including statins. Lastly, we considered season (warm: April–September versus cold: October–March) and ambient pollutants (low [<median] versus high [≥median]) including PM_2.5_, NO_2_, and O_3_ separately.

To evaluate the robustness of our findings, we performed sensitivity analyses. First, we additionally adjusted for public holidays in Sweden in our models. Second, we additionally adjusted for ambient average temperature with non-linear function. Additionally, we conducted sensitivity analyses with an alternative definition of temperature variability, calculated as the difference between daily temperature and the average temperature over the preceding 3 days. Furthermore, we adjusted PM_2.5_, NO_2_, and O_3_ separately in individual models. This approach allowed us to address potential confounding from ambient pollutants while avoiding the multicollinearity that could arise from including multiple pollutants in a single model.

The results were expressed as odds ratios (OR, with their 95% confidence intervals [CIs]) per 1 °C greater in temperature variability (1 °C increase in upward temperature shift and 1 °C decrease in downward temperature shift). We conducted all analyses using R software, version 4.3.0. Statistical significance was determined by a two-sided *p*-value of less than 0.05.

This study is reported as per the Strengthening the Reporting of Observational Studies in Epidemiology (STROBE) guideline (S1 Checklist).

## Result

### Study population and exposure data

The characteristics of the study population are presented in Tables 1 and S1 Table. The average age of the participants was 70.6 years. Among the participants, 34.5% were female and 30.3% had experienced a recurrent MI. Geographically, 47.4% of the participants were residents of the southern region of Sweden.

**Table 1:** Descriptive statistics of participant characteristics.

	Total MI (*N* = 233,617)	STEMI (*N* = 73,318)	NSTEMI (*N* = 159,679)
**Age (years)**	70.6 (12.2)	67.9 (12.3)	71.8 (11.9)
**Sex (female)**	80,564 (34.5%)	22,154 (30.2%)	58,167 (36.4%)
**Recurrent MI (yes)**	70,692 (30.3%)	–	–
**Region**			
North	33,974 (14.5%)	10,505 (14.3%)	23,410 (14.7%)
Central	88,337 (37.8%)	26,948 (36.8%)	61,300 (38.4%)
South	110,739 (47.4%)	35,707 (48.7%)	74,560 (46.7%)
**Season (cold)**	119,885 (51.3%)	37,715 (51.4%)	81,853 (51.3%)
**History of diseases**			
**Diabetes (yes)**	59,466 (25.5%)	14,412 (19.7%)	44,904 (28.1%)
**CAD (yes)**	78,592 (33.6%)	14,572 (19.9%)	63,749 (39.9%)
**Heart failure (yes)**	36,723 (15.7%)	5,493 (7.5%)	31,098 (19.5%)
**Percutaneous coronary intervention (yes)**	44,657 (19.1%)	9,080 (12.4%)	35,452 (22.2%)
**Hypertension (yes)**	138,066 (59.1%)	36,092 (49.2%)	101,618 (63.6%)
**Stroke (yes)**	26,820 (11.5%)	5,757 (7.9%)	20,987 (13.1%)
**COPD (yes)**	17,328 (7.4%)	3,821 (5.2%)	13,462 (8.4%)
**Peripheral vascular disease (yes)**	14,060 (6.0%)	2,490 (3.4%)	11,541 (7.2%)

Note: Data are reported as mean (standard deviation, SD) or *n* (%). MI, myocardial infarction; STEMI, ST-elevation myocardial infarction; NSTEMI, non-ST-elevation myocardial infarction. Total MI refers to all types of MI hospitalizations combined. Season (cold): October to March. CAD, coronary artery disease; COPD, chronic obstructive pulmonary disease.

The median level of upward temperature shift was 1.40 °C, and the median level of downward temperature shift was −1.38 °C (Table 2). The Spearman correlations between the ambient temperature variables and ambient pollutants were generally weak to moderate, ranging from −0.24 to 0.41 (S4 Fig).

**Table 2:** Descriptive statistics of the levels of ambient temperatures and ambient pollutants.

	Mean	SD	5%	25%	Median	75%	95%
**Temperature variability (°C)**							
**Upward temperature shift (°C)**	1.77	1.51	0.12	0.64	1.40	2.50	4.68
**Downward temperature shift (°C)**	−1.79	1.58	−4.84	−2.51	−1.38	−0.63	−0.12
**Daily average temperature (°C)**	6.63	7.84	−6.27	1.02	6.45	13.22	18.24
**Average of 7-day mean temperature (°C)**	6.60	7.61	−5.91	1.05	6.27	13.26	17.80
**PM**_**2.5**_ **(μg/m**^**3**^)	7.74	3.68	3.17	5.32	7.13	9.44	13.98
**NO**_**2**_ **(μg/m**^**3**^)	7.51	6.08	1.94	3.43	5.59	9.52	19.50
**O**_**3**_ **(μg/m**^**3**^)	56.00	13.88	33.89	45.85	55.68	66.20	78.46

Note: Upward temperature shift (°C): all values range from 0 to the maximum value; Downward temperature shift (°C): all values range from minimum value to 0. PM_2.5_, particulate matter with diameter ≤2.5 μm; NO_2_, nitrogen dioxide; O_3_, ozone.

### Associations of short-term exposure to temperature variability with MI hospital admissions

**Fig 2:**
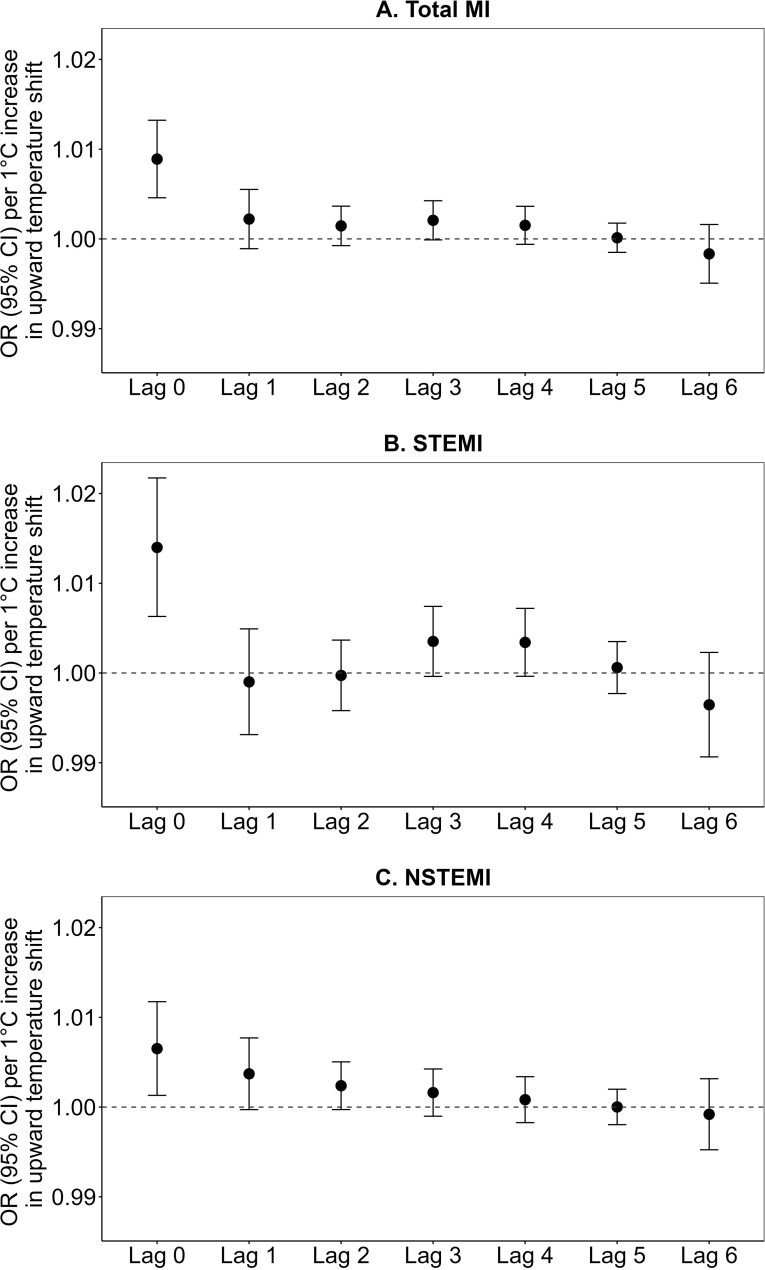
Associations of short-term exposures to upward temperature shifts (temperature variability) with (A) total MI, (B) STEMI, and (C) NSTEMI hospital admissions at lag 0–6 days. Note: MI, myocardial infarction; STEMI, ST-segment elevation myocardial infarction; NSTEMI, non-ST-segment elevation myocardial infarction. Total MI refers to all types of MI hospitalizations combined. OR, odds ratio; CI, confidence interval.

**Fig 3:**
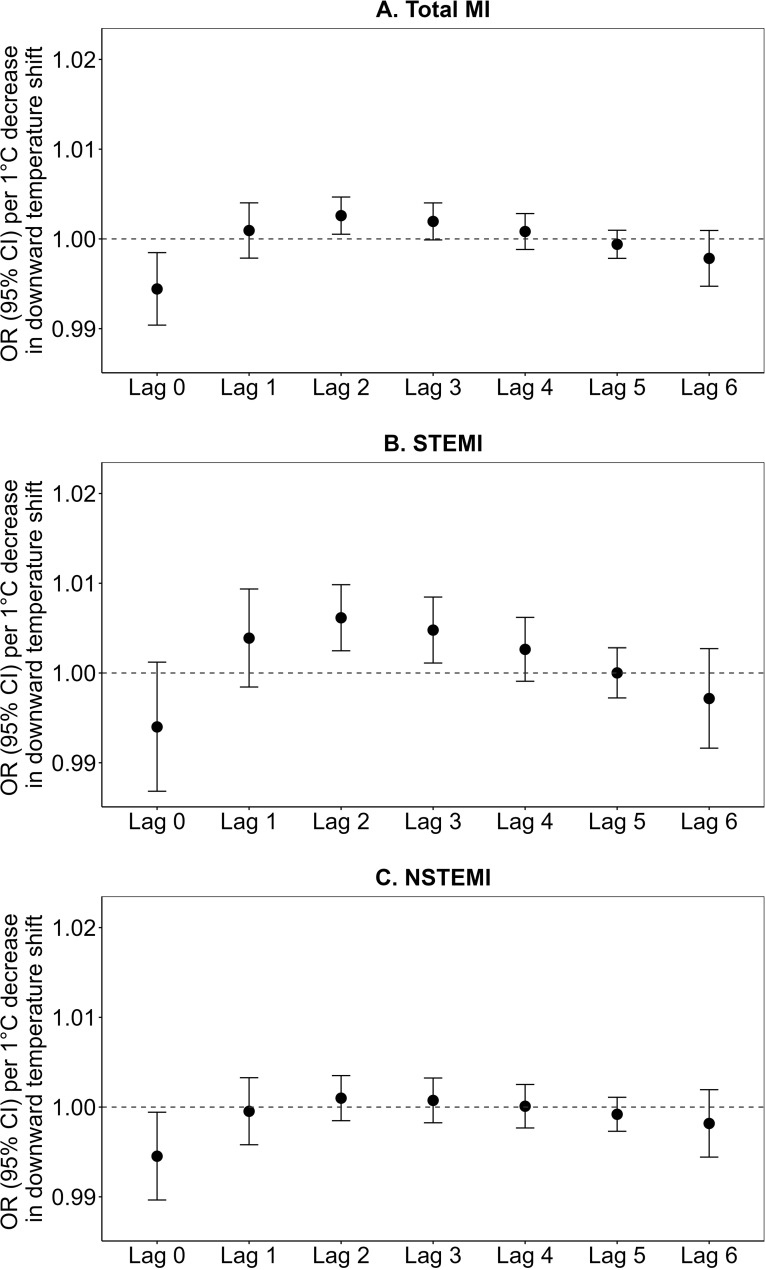
Associations of short-term exposures to downward temperature shifts (temperature variability) with (A) total MI, (B) STEMI, and (C) NSTEMI hospital admissions at lag 0–6 days. Note: MI, myocardial infarction; STEMI, ST-segment elevation myocardial infarction; NSTEMI, non-ST-segment elevation myocardial infarction. Total MI refers to all types of MI hospitalizations combined. OR, odds ratio; CI, confidence interval.

Figs 2 and 3, and S2 Table show the associations of short-term exposure to temperature variability, specifically upward and downward temperature shifts, with total MI (encompassing all MI types), STEMI, and NSTEMI hospital admissions at lags of 0–6 days. Our investigation revealed that an upward temperature shift was associated with increased risks of total MI (encompassing all MI types), STEMI, and NSTEMI at lag 0 day, with ORs (95% CI) of 1.009 (1.005, 1.013; *p* < 0.001), 1.014 (1.006, 1.022; *p* < 0.001), and 1.007 (1.001, 1.012; *p* = 0.014) per 1 °C increase, respectively (Fig 2 and S2 Table). Those associations exhibited attenuation and became statistically non-significant over subsequent lag periods, ranging from lag 1 to 6 days.

Furthermore, a downward temperature shift, calculated as the absolute value of the negative difference between the temperature on the day of the MI event and the average temperature of the preceding week, was associated with increased risks of total MI hospital admissions (encompassing all MI types) at a lag of 2 days with an OR (95% CI): 1.003 (1.001, 1.005; *p* = 0.014), and STEMI hospital admissions at lags 2 and 3 days with OR (95% CI): 1.006 (1.002, 1.010; *p* = 0.001) and 1.005 (1.001, 1.008; *p* = 0.011), per 1 °C decrease, respectively (Fig 3 and S2 Table). In contrast, higher downward temperature shifts were associated with decreased risks of total MI (encompassing all MI types) and NSTEMI hospital admissions at lag 0 day. No significant associations were observed between downward temperature shifts and MI at other lag days.

Our analysis uncovered specific lag periods for the significant associations between temperature variability and MI risk. Upward temperature shifts showed a significant association with an increased risk of MI hospital admissions at lag 0 day, while downward temperature shifts demonstrated a peak association with an increased risk of MI hospital admissions at lag 2 day. Consequently, we focused on these specific lags—lag 0 for upward temperature shifts and lag 2 for downward temperature shifts—as the main findings for further analyses.

**Fig 4:**
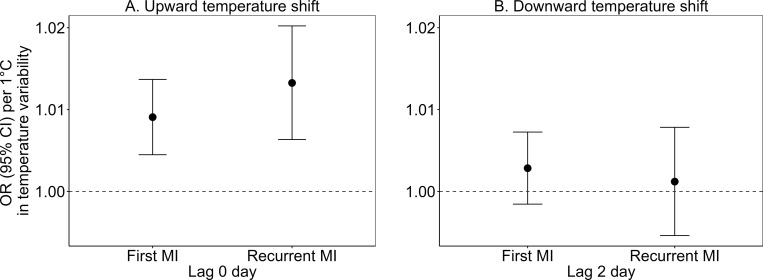
Associations of short-term temperature variability exposure with the first and the recurrent MI hospitalizations: (A) upward temperature shifts at lag 0 day and (B) downward temperature shifts at lag 2 days. Note: MI, myocardial infarction; OR, odds ratio; CI, confidence interval.

We also found an association between upward temperature shift and increased risk of hospital admissions for both first and recurrent MIs at lag 0 day, with OR (95% CI) of 1.009 (1.004, 1.014; *p* < 0.001) and 1.013 (1.006, 1.020; *p* < 0.001), per 1 °C increase, respectively (Fig 4 and S3 Table). There was a suggestion of a stronger association among recurrent MIs, but the difference was not statistically significant compared to the first MI. We observed positive, but non-significant, associations between higher downward temperature shifts and risks of hospital admissions for both first and recurrent MI at lag 2 day.

**Fig 5:**
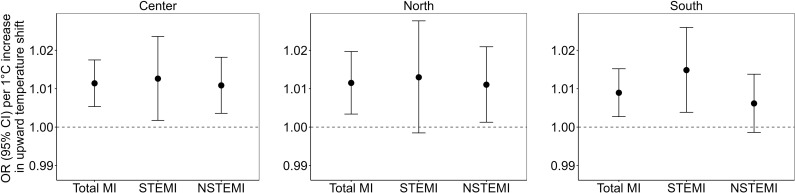
Associations of upward temperature shifts (temperature variability) exposure at lag 0 day with total MI, STEMI, and NSTEMI hospitalizations in the central, southern, and northern regions of Sweden. Note: MI, myocardial infarction; STEMI, ST-segment elevation myocardial infarction; NSTEMI, non-ST-segment elevation myocardial infarction. Total MI refers to all types of MI hospitalizations combined. OR, odds ratio; CI, confidence interval.

In our regional analyses (Fig 5 and S4 Table), we observed that higher upward temperature shift at lag 0 day was associated with increased risks of total MI (encompassing all MI types), STEMI, or NSTEMI hospital admissions in the southern, central, and northern parts of Sweden, with no distinct difference in the effect estimates between regions. Moreover, while significant associations between the downward temperature shift at lag 2 day and an increased risk of STEMI were only found in central Sweden, the effect estimates between regions showed no statistically significant differences (S5 Fig and S4 Table).

### Effect modification

**Fig 6:**
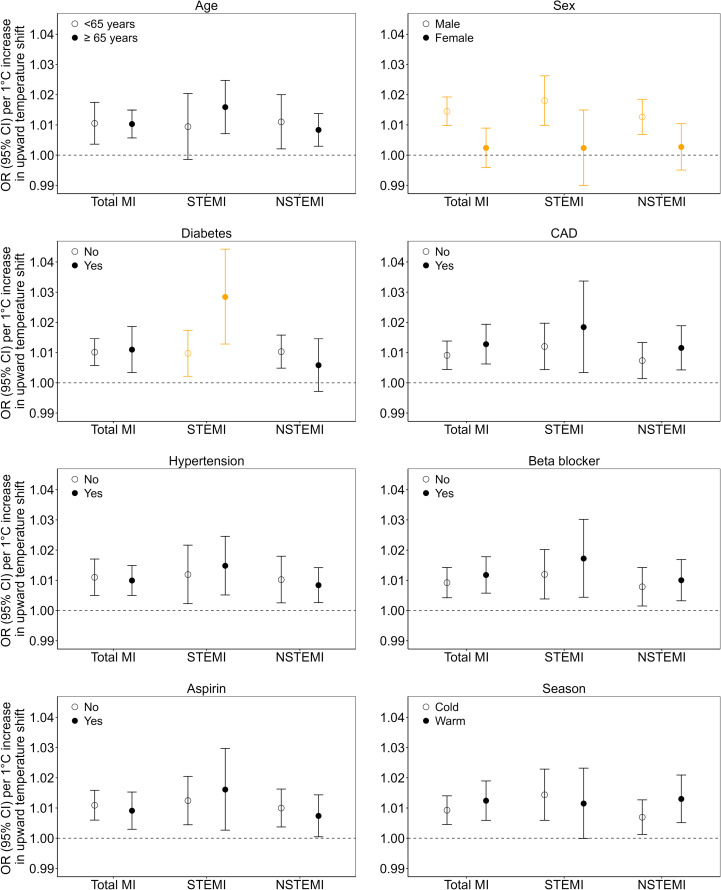
Effect modifications of upward temperature shift (temperature variability) on MI hospital admissions by modifiers at lag 0 day. Note: Orange error bars represent *p*-value for the interaction term <0.05. MI, myocardial infarction; STEMI, ST-segment elevation myocardial infarction; NSTEMI, non-ST-segment elevation myocardial infarction. Total MI refers to all types of MI hospitalizations combined. CAD, coronary artery disease. Season: cold, October to March; warm, April to September. OR, odds ratio; CI, confidence interval.

We found that upward temperature shifts had a significantly stronger association with total MI (encompassing all MI types), STEMI, and NSTEMI hospital admissions in males than in females (Fig 6). Similarly, individuals with a history of diabetes displayed a stronger association between upward temperature shift and STEMI hospital admissions than those without diabetes. However, we did not observe effect modifications for other pre-existing conditions (CAD, heart failure, hypertension, percutaneous coronary intervention, stroke, COPD, and peripheral vascular disease), demographic and lifestyle factors, medication history (any medications, heart medications, anti-hypertensive medications, anticoagulant medications, anti-diabetic medications, and lipid-lowering medications), season, or ambient pollutants (Figs 6 and S6, S7, and S8).

In addition, we found that downward temperature shifts were significantly more strongly associated with total MI (encompassing all MI types) and NSTEMI hospital admissions in patients exposed during the cold season compared to the warm season (S9 Fig). The association between downward temperature shift and MI hospital admissions was generally unmodified by the other investigated modifiers, except for a stronger association with total MI (encompassing all MI types) and NSTEMI hospital admissions in patients not using long-acting nitro compared to those who did (S10, S11, and S12 Figs).

### Sensitivity analysis

Upward temperature shifts remained robustly associated with increased risk of MI hospital admissions even after adjusting for public holiday, average temperature, and air pollutants (PM_2.5_, NO_2_, and O_3_), as well as employing alternative definitions of temperature variability, defined as the difference between daily temperature and the average temperature over the preceding 3 days (S13 Fig). Downward temperature shifts generally maintained consistent associations with MI risk, except for the association appeared to be attenuated when additionally adjusted for average temperature (S14 Fig).

## Discussion

In this comprehensive, nationwide, time-stratified, case-crossover study, we found that higher temperature variability, characterized by greater upward or downward temperature shifts, was associated with increased risks of MI hospitalizations. Specifically, an upward temperature shift was associated with increased risks of total MI (encompassing all MI types), STEMI, and NSTEMI hospitalizations at a lag of 0 day. Moreover, downward temperature shifts exhibited a delayed association, with increased risks of total MI at lag 2 days and STEMI at lag 2 and 3 days.

Although an increasing body of research has investigated the relationship between non-optimal temperatures and MI [8–12], research on temperature variability and MI occurrence or hospital admissions remains limited, with mixed findings [14–26]. A city-based study in Beijing, China, which uniquely explored directional temperature variability, found that daily mean temperature differences at the first percentile (−6 °C) and 99th percentile (5 °C) were associated with increased MI hospitalizations, compared to 1.4 °C [25]. However, this study did not differentiate between MI subtypes. A study applying the SWEDEHEART database from 1998 to 2013 reported a significant association between higher diurnal temperature range and an increased risk of total MI and NSTEMI hospitalizations, but not STEMI in Sweden [24]. Similar findings for diurnal temperature range or interday temperature differences and MI occurrence were reported [19,21,23,26]. Research from Japan on MI yielded conflicting results regarding the association with temperature changes [14–18]. Some studies found no association between diurnal temperature range and MI occurrence or hospital admissions [20,22]. However, with the exception of one study that examined temperature differences at the 1st, 5th, 95th, and 99th percentile [25], none of prior studies distinguished between upward and downward temperature shifts, limiting our understanding of the directional associations of temperature variability with MI occurrence or hospital admissions risk. Additionally, no studies have investigated associations of short-term exposures to directional temperature variability with MI and its specific subtypes of STEMI and NSTEMI hospital admissions within the same population.

Our study also found significant associations between exposure to sudden increases in daily temperature in comparison with the preceding weeks’ average temperature, or upward temperature shift as we called it, and increased risks of both first and recurrent MI hospitalizations. This finding suggests that temperature variability, sudden upward swings or spikes in temperature, may precipitate MI events in lower and higher-risk populations. Interestingly, the associations between upward temperature shifts and MI hospitalization were slightly stronger in patients who experienced recurrent MI compared to those with their first MI, although this difference did not reach a statistical significance. This suggests a potential trend toward greater vulnerability among patients with recurrent MI, which warrants further investigation. The presence of patients with recurrent MI may also influence the accuracy of risk estimates in the overall population, indicating that future studies should consider stratifying analyses by first and recurrent MI events to better understand the differential impacts of temperature variability on these populations. Additionally, we observed consistent associations between upward temperature shift and elevated risks of MI in the southern, central, and northern parts of Sweden. The association was robust across these distinct geographic locations, lending further weight to the nationwide relationships. This indicates susceptibility to upward temperature swings irrespective of geographic location in Sweden, highlighting the broad public health impacts of temperature variability.

Our study found that each 1 °C greater in temperature variability was associated with a 0.3%–1.4% increase in MI hospitalization risk. Although these effect sizes may appear modest at the individual level, their implications become more substantial when considered in the context of population health and more extreme temperature shifts. Unlike clinical interventions, environmental exposures affect entire populations continuously, and small daily risks can accumulate over time, leading to significant long-term health burdens. Temperature shifts often exceed 1 °C, e.g., a 5 °C temperature shift, which is not uncommon in many regions, would correspond to approximately a 1.5%−7% increase in MI risk according to our findings. This magnification with larger temperature variations underscores the potential impact of extreme weather events on cardiovascular health. Moreover, given that temperature variability affects entire populations simultaneously, even small individual-level effects can translate to a considerable number of additional MI cases when applied to an entire population and substantial public health impacts. Contextualizing our results with other environmental risk factors, a study found that each 1 μg/m^3^ increase in O_3_ was associated with a 0.075% increase in MI risk [38], indicating that our observed association for temperature shifts is comparatively larger. These underscore the importance of considering temperature variability in public health strategies, particularly in the context of increasing climate variability.

Furthermore, our findings suggest that exposure to upward temperature shift has a stronger association with MI hospitalizations risk among males than females. The more pronounced impact of temperature variability on men may be attributed to sex differences in behavior, physiology, and thermoregulation [39]. For example, males have been observed to experience lower thermal sensation and reduced warm sensitivity compared to females [39]. When exposed to sudden upward temperature shifts, males may be less sensitive to these thermal cues compared to females, potentially leading to delayed behavioral responses, such as seeking cooler environments or modifying clothing, thereby increasing their vulnerability to upward temperature shift—adverse health outcomes. Conversely, the heightened thermal sensitivity observed in females might confer a protective advantage by prompting earlier thermal behavior modifications, thereby potentially mitigating the adverse associations with sudden temperature increases compared to males. Additionally, patients with a history of diabetes appeared more vulnerable to the associations between the upward temperature swings and the risk of MI hospitalizations. This observation might be attributed to impaired endothelial function and diminished skin blood flow among individuals with diabetes, thereby limiting their ability to sweat and regulate body temperature effectively in high-temperature exposures [40]. Regarding downward temperature shifts, we observed a significantly stronger association on MI in patients exposed during the cold season compared to the warm season. This finding aligns with our expectation that during the cold season, particularly in regions with colder climates like Sweden, the human body is already under greater thermoregulatory stress [41]. A further downward shift in temperature may overwhelm the body’s adaptive mechanisms, exerting additional stress on the cardiovascular system more readily during the cold season.

Contrary to our initial hypothesis, downward temperature shifts showed associations with lowered risks of total MI (encompassing all MI types) and NSTEMI hospitalizations at lag 0 day. To our knowledge, this is one of the few studies examining directional temperature variability separately (upward and downward temperature shifts) in Sweden, the interpretation for this unexpected finding is less defined. Only one study that examined the directional temperature variability in China also found that neighboring-day mean temperature difference at the fifth percentile was associated with a decreased risk of MI at lag 0 day, though the association was not statistically significant [25]. The observed protective association of downward temperature shifts at lag 0 aligns with findings from prior studies indicating that lower average temperatures at lag 0 may be associated with reduced risk of MI [42–44]. However, the underlying mechanisms through which lower temperatures or downward temperature shifts might be associated with a reduced risk of MI at lag 0 day remain unclear and warrant further exploration. Notably, this significant association was observed exclusively in the southern region of Sweden, characterized by comparatively warmer temperatures than its central and northern counterparts, in regional analyses (S15 Fig). A possible explanation for this finding could be that in the southern regions, a downward shift in temperature may provide relief from the heat, contrasting with our observation of increased MI risk associated with upward temperature shifts at lag 0 day, potentially reducing the physiological strain associated with high temperatures. This unexpected finding highlights the complex nature of temperature-health relationships, further research is essential to validate these initial findings.

Although our study focuses solely on Sweden, the country shares similarities with other northern European countries, such as Norway, in terms of healthcare systems, climate patterns, and population demographics. These similarities support the generalizability of our findings to at least northern European countries. However, local variations in population adaptation, urban infrastructure, and environmental policies should be considered when applying these results to other settings.

Although our approach of assessing temperature variability by comparing same-day temperatures to the preceding 7-day average temperatures may not be immediately intuitive for individual use, it captures multiday trends relevant to human acclimatization and provides insights into the health impacts of temperature fluctuations. Although individuals are unlikely to compute 7-day average temperature themselves, our findings can inform early warning systems, where meteorological and public health agencies integrate this metric into forecasting models to identify high-risk periods and issue timely warnings without requiring individuals to calculate their own risk. Public health messaging could then target vulnerable populations during significant temperature shifts, and healthcare systems could use these insights to anticipate MI cases and allocate resources more effectively. Although 7-day average temperature is not yet widely accessible to the public in Sweden, our research may highlight the potential for meteorological services to incorporate this ambient temperature variability metric into forecasts, contributing to more effective public health interventions to reduce temperature-related cardiovascular morbidity.

Understanding how temperature variability impacts MI through its underlying mechanisms is still unclear. Previous studies reported that short-term exposure to temperature variability significantly correlates with increased C-reactive protein levels [45]. This suggests that inflammation could be a biological mechanism underlying the observed associations between temperature variability and cardiovascular outcomes [45,46]. Moreover, exposure to sudden increases or spikes in temperature induce physiological perturbations, including dehydration, electrolyte dysregulation, and hemoconcentration [47–49]. These physiological changes can precipitate sympathetic activation and tachycardia, potentially culminating in demand-related ischemia or atherosclerotic plaque destabilization [47–49]. Conversely, exposure to downward temperature shifts may trigger sympathetic stimulation, peripheral vasoconstriction, and enhanced muscle tonicity, thereby elevating blood pressure, and promote cholesterol crystal deposition within atheromatous lesions [47–49]. Additionally, abrupt upward and downward temperature shifts within a short timeframe can potentially disrupt personal thermoregulation strategies, rendering the body inadequately equipped to cope with temperature changes [50–52].

Our study has several limitations. This study relies on outdoor ambient temperature measurements rather than personal exposures, which may increase the likelihood of misclassification of exposures. However, it is important to note that our study focuses on short-term associations, which are more dependent on day-to-day variations in temperature rather than the actual temperature levels. Moreover, in the context of large-scale data collection, the task of monitoring personal temperature levels for each participant is pragmatically unfeasible. Therefore, despite its potential limitations, the utilization of ambient temperature data emerges as the most practical approach currently available for studies involving extensive sample sizes. Second, some patients with MI may die before reaching the hospital, potentially introducing a selection bias in the analysis of hospitalized patients. Third, our study did not account for relative humidity due to a lack of access to data, which may influence cardiovascular health outcomes. Fourth, our time-stratified, case-crossover study design may not fully capture the displacement associations over longer periods. Future research using different study designs, such as cohort studies with extended lag periods, could confirm our findings*.* Despite these limitations, our findings provide insights into temperature variability and risk of MI hospital admission, suggesting areas for future research. The definition of temperature variability itself warrants further examination. Although our study used a specific measure of variability, future research could investigate alternative definitions or metrics to capture different aspects of temperature fluctuation and their respective impacts on MI risk. Geographical comparisons, such as urban versus rural areas, could highlight differences in vulnerability, informing targeted interventions. Importantly, future research should also focus on elucidating the underlying physiological mechanisms by which temperature variability affects cardiovascular health. These directions will deepen our understanding of climate variability’s impact on health and guide effective public health policies amid climate change.

In conclusion, this comprehensive, nationwide study contributes insights that short-term exposures to higher temperature variability—greater upward or downward temperature shifts—are associated with an increased risk of hospitalization for MI. This finding highlights the cardiovascular health threats posed by higher temperature variability, which are anticipated to intensify in prevalence due to climate change.

## Supporting information

S1 Checklist STROBE Checklist. (DOCX)

S1 Text Formulation of the conditional logistic regression model with distributed lag non-linear model (DLNM). (DOCX)

S2 Text Explanation of the *z*-test for effect Modification. (DOCX)

S1 Table Descriptive statistics of participants’ characteristics and medication usage. Note: Data are reported as mean (standard deviation, SD) or *n* (%). Education, low, indicating education up to high school level or less. MI, myocardial infarction; STEMI, ST-segment elevation myocardial infarction; NSTEMI, non-ST-segment elevation myocardial infarction. Total MI refers to all types of MI hospitalizations combined. ACE, angiotensin-converting enzyme inhibitors. A2 blockers, angiotensin II receptor blockers. DOAC, including apixaban, dabigatran etexilate, edoxaban, and rivaroxaban.
(DOCX)

S2 Table Associations of short-term exposures to temperature variability (upward and downward temperature shifts) with MI hospitalizations. Note: MI, myocardial infarction; STEMI, ST-segment elevation myocardial infarction; NSTEMI, non-ST-segment elevation myocardial infarction. Total MI refers to all types of MI hospitalizations combined. OR, odds ratio; CI, confidence interval.
(DOCX)

S3 Table Associations of short-term exposures to temperature variability (upward and downward temperature shifts) with first and recurrent MI hospitalizations. Note: MI, myocardial infarction; OR, odds ratio; CI, confidence interval.
(DOCX)

S4 Table Associations of upward temperature shifts at lag 0 day and downward temperature shifts at lag 2 day with total MI, STEMI, and NSTEMI hospitalizations in the central, southern, and northern regions of Sweden. Note: MI, myocardial infarction; STEMI, ST-segment elevation myocardial infarction; NSTEMI, non-ST-segment elevation myocardial infarction. Total MI refers to all types of MI hospitalizations combined. OR, odds ratio; CI, confidence interval.
(DOCX)

S1 Fig Yearly average of mean temperature trends in Sweden (2005–2019). (DOCX)

S2 Fig Flowchart of data management. Note: MI, myocardial infarction; STEMI, ST-segment elevation myocardial infarction; NSTEMI, non-ST-segment elevation myocardial infarction.
(DOCX)

S3 Fig Exposure–response functions of temperature variability and total MI, STEMI, and NSTEMI. Note: LR-test, likelihood ratio test (temperature variability was included as natural cubic spline with 3dfs versus temperature variability was included as linear). MI, myocardial infarction; STEMI, ST-segment elevation myocardial infarction; NSTEMI, non-ST-segment elevation myocardial infarction. Total MI refers to all types of MI hospitalizations combined.
(DOCX)

S4 Fig Spearman correlations between the ambient temperatures and ambient pollutants variables. Note: PM_2.5_, particulate matter with diameter ≤2.5 micrometers. NO_2_, nitrogen dioxide; O_3_, ozone.
(DOCX)

S5 Fig Associations of downward temperature shifts (temperature variability) at lag 2 day with total MI, STEMI, and NSTEMI hospitalizations in the central, southern, and northern regions of Sweden. Note: MI, myocardial infarction; STEMI, ST-segment elevation myocardial infarction; NSTEMI, non-ST-segment elevation myocardial infarction. Total MI refers to all types of MI hospitalizations combined. OR, odds ratio; CI, confidence interval.
(DOCX)

S6 Fig Effect modifications of upward temperature shift (temperature variability) on MI hospital admissions by history of diseases, smoking status, and education at lag 0 day. Note: MI, myocardial infarction; STEMI, ST-segment elevation myocardial infarction; NSTEMI, non-ST-segment elevation myocardial infarction; COPD, chronic obstructive pulmonary disease. Total MI refers to all types of MI hospitalizations combined. OR, odds ratio; CI, confidence interval.
(DOCX)

S7 Fig Effect modifications of upward temperature shift on MI hospital admissions by history of medications at lag 0 day. Note: MI, myocardial infarction; STEMI, ST-segment elevation myocardial infarction; NSTEMI, non-ST-segment elevation myocardial infarction. Total MI refers to all types of MI hospitalizations combined. ACE inhibitors, angiotensin-converting enzyme inhibitors. A2 blockers, angiotensin II receptor blockers. DOAC, including apixaban, dabigatran etexilate, edoxaban, and rivaroxaban. OR, odds ratio; CI, confidence interval.
(DOCX)

S8 Fig Effect modifications of upward temperature shift on MI hospital admissions by air pollutants at lag 0 day. Note: MI, myocardial infarction; STEMI, ST-segment elevation myocardial infarction; NSTEMI, non-ST-segment elevation myocardial infarction. Total MI refers to all types of MI hospitalizations combined. PM_2.5_, particulate matter with diameter ≤2.5 micrometers. NO_2_, nitrogen dioxide; O_3_, ozone; OR, odds ratio; CI, confidence interval.
(DOCX)

S9 Fig Effect modifications of downward temperature shift on MI hospital admissions by modifiers at lag 2 day. Note: Red error bars represent *p*-value for the interaction term < 0.05. MI, myocardial infarction; STEMI, ST-segment elevation myocardial infarction; NSTEMI, non-ST-segment elevation myocardial infarction. Total MI refers to all types of MI hospitalizations combined. Season: cold, October to March; warm, April to September. OR, odds ratio; CI, confidence interval.
(DOCX)

S10 Fig Effect modifications of downward temperature shift on MI hospital admissions by history of diseases at lag 2 day. Note: MI, myocardial infarction; STEMI, ST-segment elevation myocardial infarction; NSTEMI, non-ST-segment elevation myocardial infarction. Total MI refers to all types of MI hospitalizations combined. CAD, coronary artery disease. COPD, chronic obstructive pulmonary disease; OR, odds ratio; CI, confidence interval.
(DOCX)

S11 Fig Effect modifications of downward temperature shift on MI hospital admissions by history of medications at lag 2 day. Note: Red error bars represent *p*-value for the interaction term <0.05. MI, myocardial infarction; STEMI, ST-segment elevation myocardial infarction; NSTEMI, non-ST-segment elevation myocardial infarction. Total MI refers to all types of MI hospitalizations combined. ACE inhibitors, angiotensin-converting enzyme inhibitors. A2 blockers, angiotensin II receptor blockers. DOAC, including apixaban, dabigatran etexilate, edoxaban, and rivaroxaban. OR, odds ratio; CI, confidence interval.
(DOCX)

S12 Fig Effect modifications of downward temperature shift on MI hospital admissions by air pollutants at lag 2 day. Note: MI, myocardial infarction; STEMI, ST-segment elevation myocardial infarction; NSTEMI, non-ST-segment elevation myocardial infarction. Total MI refers to all types of MI hospitalizations combined. PM_2.5_, particulate matter with diameter ≤2.5 micrometers. NO_2_, nitrogen dioxide; O_3_, ozone; OR, odds ratio; CI, confidence interval.
(DOCX)

S13 Fig Sensitivity analyses: Associations of upward temperature shifts (temperature variability) at lag 0 day with (A) total MI, (B) STEMI, and (C) NSTEMI hospital admissions. Note: MI, myocardial infarction; STEMI, ST-segment elevation myocardial infarction; NSTEMI, non-ST-segment elevation myocardial infarction. Total MI refers to all types of MI hospitalizations combined. PM_2.5_, particulate matter with diameter ≤2.5 micrometers. NO_2_, nitrogen dioxide; O_3_, ozone; OR, odds ratio; CI, confidence interval.
(DOCX)

S14 Fig Sensitivity analyses: Associations of downward temperature shifts (temperature variability) at lag 2 day with (A) total MI, (B) STEMI, and (C) NSTEMI hospital admissions. Note: MI, myocardial infarction; STEMI, ST-segment elevation myocardial infarction; NSTEMI, non-ST-segment elevation myocardial infarction. Total MI refers to all types of MI hospitalizations combined. PM_2.5_, particulate matter with diameter ≤2.5 micrometers. NO_2_, nitrogen dioxide; O_3_, ozone; OR, odds ratio; CI, confidence interval.
(DOCX)

S15 Fig Associations of downward temperature shifts at lag 0 day with total MI, STEMI, and NSTEMI hospital admissions in central, southern, and northern regions of Sweden. Note: MI, myocardial infarction; STEMI, ST-segment elevation myocardial infarction; NSTEMI, non-ST-segment elevation myocardial infarction. Total MI refers to all types of MI hospitalizations combined. OR, odds ratio; CI, confidence interval.
(DOCX)

## Abbreviations

A2 blockers: angiotensin II receptor blockers
ACE: angiotensin-converting enzyme
BMI: body mass index
CAD: coronary artery disease
CI: confidence interval
COPD: chronic obstructive pulmonary disease
Dfb: humid continental climate
Dfc: subarctic climate
DLNM: distributed lag non-linear model
EU: European Union
ICD-10: International Classification of Diseases, 10th Revision
LST&E: land surface temperature and emissivity
LST: land surface temperature
MI: myocardial infarction
NO_2_: Nitrogen dioxide
NSTEMI: non-ST-segment elevation myocardial infarction
O_3_: Ozone
OR: odds ratio
PM_2.5_: particulate matter with a diameter less than 2.5μm
SD: standard deviation
SMHI: Swedish Meteorological and Hydrological Institute
STEMI: ST-segment elevation myocardial infarction
STROBE: Strengthening the Reporting of Observational Studies in Epidemiology
