# Supplementary material for: Short-term exposure to ambient temperature variability and myocardial infarction hospital admissions: A nationwide case-crossover study in Sweden
Source: PLoS Med. 2025 May 20;22(5):e1004607. doi: 10.1371/journal.pmed.1004607 (PMC12091774; doi:10.1371/journal.pmed.1004607)
Supplement: S1 Text — (DOCX) [file pmed.1004607.s002.docx]

### **Text S1. Formulation of the conditional logistic regression model with distributed lag non-linear model (DLNM)**

**The “crossbasis” function in the DLNM framework is defined as:**

$${cb}_{lag 0-6} =\sum_{l=0}^{L} f\cdot w ({TV}_{i, t-l}, l)$$

where:

**Exposure-Response:** $f({TV}_{i, t-l})=\mathrm{thr}\left( {TV}_{i, t-l}\mathbf{}\mathbf{,}thr.value=0 \right)$

**Lag-Response:** $w (l)= ns (l,knots=logknots(L,2))$

In this context, *cb_lag 0-6_* is the “crossbasis” function capturing both exposure-response with *f (TV_i, t-l_)* and lag-response with *w (l)* dimensions^1^. The exposure-response relationship modeled using a threshold function (*thr (TV_i_, _t-l_), thr.value=0*) with a threshold value of 0 to differentiate between upward and downward temperature shifts, and the lag-response relationship modeled using a natural cubic spline (ns) with two interior knots at evenly spaced log values of lag days (0 to 6 days, *ns (l, knots = logknots (L,2*)). *TV_i, t-l_* is the temperature variability for individual *i* at time *t−l*, where *l*represents the lag period (ranging from 0 to *L*=6 days). *L* is the maximum lag (in this case, 6 days).

**The conditional logistic regression model incorporating the DLNM is expressed as:**

$${\log\left( \frac{P \left( Y_{\mathrm{it}}=1 \right)}{1-P (Y_{\mathrm{it}}=1)} \right) =\alpha+\beta_{1}\cdot{cb}_{lag 0-6} +stratum}_{i}$$

Here*, Y_it_​* is the binary outcome variable indicating whether individual *i* experienced an MI hospitalization at time *t* (1 = MI hospitalization, 0 = no MI hospitalization). *P (Y_it​_ =1*) is the probability of MI hospitalization for individual *i* at time *t*. *α* is the intercept term. *β*_1_​ is the coefficient representing the effect of temperature variability on MI hospitalization. *stratum_i​_* is the stratum-specific intercept, which accounts for the matched case-control structure in the time-stratified case-crossover design.

# **Reference**

1. Gasparrini A, Scheipl F, Armstrong B, Kenward MG. A penalized framework for distributed lag non-linear models. *Biometrics.* 2017;73(3):938-948.
