# Supplementary material for: Short-term exposure to ambient temperature variability and myocardial infarction hospital admissions: A nationwide case-crossover study in Sweden
Source: PLoS Med. 2025 May 20;22(5):e1004607. doi: 10.1371/journal.pmed.1004607 (PMC12091774; doi:10.1371/journal.pmed.1004607)
Supplement: S2 Text — (DOCX) [file pmed.1004607.s003.docx]

### **Text S2. Explanation of the z-test for effect Modification**

The z-test was employed to evaluate whether the effect of temperature variability on MI hospitalizations differed significantly between subgroups. The test statistic z quantifies the difference in effect sizes (log-odds ratios) between the two subgroups, standardized by the combined uncertainty (standard errors) of the estimates. A significant z-score (∣z∣>1.96 for α=0.05) indicates a statistically significant difference in the effect of temperature variability between the subgroups. The test statistic z is calculated as follows:

$$\text{z =}\frac{\log\left( \text{OR}_{1} \right)\text{-}\log\left( \text{OR}_{2} \right)}{\sqrt{{{SE}_{1}}^{\text{2}}\text{+ }{{SE}_{2}}^{\text{2}}}}$$

where log (OR_1_) and log (OR_2_) represent the natural logarithms of the odds ratios for the two subgroups under comparison. The terms SE_1_ and SE_2_ are the standard errors of the log-transformed odd ratios for each subgroup, derived from the respective confidence intervals of each subgroup.
