# Supplementary material for: Short-term exposure to ambient temperature variability and myocardial infarction hospital admissions: A nationwide case-crossover study in Sweden
Source: PLoS Med. 2025 May 20;22(5):e1004607. doi: 10.1371/journal.pmed.1004607 (PMC12091774; doi:10.1371/journal.pmed.1004607)
Supplement: S1 Table — Note: Data are reported as mean (standard deviation, SD) or n (%). Education, low, indicating education up to high school level or less. MI, myocardial infarction; STEMI, ST-segment elevation myocardial infarction; NSTEMI, non-ST-segment elevation myocardial infarction. Total MI refers to all types of MI hospitalizations combined. ACE, angiotensin-converting enzyme inhibitors. A2 blockers, angiotensin II receptor blockers. DOAC, including apixaban, dabigatran etexilate, edoxaban, and rivaroxaban. (DOCX) [file pmed.1004607.s004.docx]

### **Table S1.** **Descriptive statistics of participants’ characteristics and medication usage**

|  | **Total MI (N=233,617)** | **STEMI (N=73,318)** | **NSTEMI (N=159,679)** |
| --- | --- | --- | --- |
| **Age (≥65 years)** | 162222 (69.4 %) | 44698 (61.0 %) | 117075 (73.3 %) |
| **BMI (kg/m²)** | 27.1 (5.9) | 27.0 (5.7) | 27.2 (6.0) |
| **Smoking status** |  |  |  |
| Never | 93037 (39.8 %) | 27534 (37.6 %) | 65238 (40.9 %) |
| Previous smoker | 78530 (33.6 %) | 22050 (30.1 %) | 56340 (35.3 %) |
| Smoke smoker | 46692 (20.0 %) | 19846 (27.1 %) | 26734 (16.7 %) |
| **Education (low)** | 188750 (80.8 %) | 58560 (79.9 %) | 129663 (81.2 %) |
| **History of medications** |  |  |  |
| **Any medications (yes)** | 164366 (70.4 %) | 41926 (57.2 %) | 121961 (76.4 %) |
| **Any heart medications (yes)** | 141618 (60.6 %) | 33954 (46.3 %) | 107238 (67.2 %) |
| **ACE inhibitor (yes)** | 53026 (22.7 %) | 11820 (16.1 %) | 40991 (25.7 %) |
| **A2 blocker (yes)** | 39241 (16.8 %) | 9611 (13.1 %) | 29587 (18.5 %) |
| **Beta blocker (yes)** | 93723 (40.1 %) | 20249 (27.6 %) | 73138 (45.8 %) |
| **Diuretic (yes)** | 57092 (24.4 %) | 11169 (15.2 %) | 45732 (28.6 %) |
| **Aldosterone inhibitor (yes)** | 4466 (1.9 %) | 750 (1.0 %) | 3716 (2.3 %) |
| **Digoxin (yes)** | 4692 (2.0 %) | 781 (1.1 %) | 3893 (2.4 %) |
| **Long acting nitro (yes)** | 31530 (13.5 %) | 3858 (5.3 %) | 27555 (17.3 %) |
| **Any anti-hypertensive medications (yes)** | 146280 (62.6 %) | 35879 (48.9 %) | 109971 (68.9 %) |
| **Calcium channel blocker (yes)** | 46666 (20.0 %) | 11701 (16.0 %) | 34862 (21.8 %) |
| **Any anticoagulant medications (yes)** | 107609 (46.1 %) | 21371 (29.1 %) | 85849 (53.8 %) |
| **Aspirin (yes)** | 89000 (38.1 %) | 18084 (24.7 %) | 70596 (44.2 %) |
| **Oral anticoagulant (yes)** | 16347 (7.0 %) | 2815 (3.8 %) | 13459 (8.4 %) |
| **DOAC (yes)** | 4003 (1.7 %) | 720 (1.0 %) | 3283 (2.1 %) |
| **Platelet inhibitor (yes)** | 21305 (9.1 %) | 3288 (4.5 %) | 17861 (11.2 %) |
| **Clopidogrel (yes)** | 16283 (7.0 %) | 2461 (3.4 %) | 13668 (8.6 %) |
| **Anti-diabetic medications (yes)** | 44366 (19.0 %) | 9887 (13.5 %) | 34371 (21.5 %) |
| **Any lipid-lowering medications (yes)** | 78692 (33.7 %) | 16326 (22.3 %) | 62076 (38.9 %) |
| **Statin (yes)** | 76597 (32.8 %) | 15896 (21.7 %) | 60413 (37.8 %) |

Note: Data are reported as mean (standard deviation, SD) or n (%). Education, low, indicating education up to high school level or less. MI, myocardial infarction. STEMI, ST-segment elevation myocardial infarction. NSTEMI, non-ST-segment elevation myocardial infarction. Total MI refers to all types of MI hospitalizations combined. ACE: angiotensin-converting enzyme inhibitors. A2 blockers, angiotensin II receptor blockers. DOAC, including apixaban, dabigatran etexilate, edoxaban, and rivaroxaban.
