# Supplementary material for: Short-term exposure to ambient temperature variability and myocardial infarction hospital admissions: A nationwide case-crossover study in Sweden
Source: PLoS Med. 2025 May 20;22(5):e1004607. doi: 10.1371/journal.pmed.1004607 (PMC12091774; doi:10.1371/journal.pmed.1004607)
Supplement: S2 Table — Note: MI, myocardial infarction; STEMI, ST-segment elevation myocardial infarction; NSTEMI, non-ST-segment elevation myocardial infarction. Total MI refers to all types of MI hospitalizations combined. OR, odds ratio; CI, confidence interval. (DOCX) [file pmed.1004607.s005.docx]

### **Table S2. Associations of short-term exposures to temperature variability (upward and downward temperature shifts) with MI hospital admissions.**

| **Temperature variability** | **Lag** | **Total MI** | |  | **STEMI** | |  | **NSTEMI** | |
| --- | --- | --- | --- | --- | --- | --- | --- | --- | --- |
|  |  | **OR (95% CI)** | ***p*-value** |  | **OR (95% CI)** | ***p*-value** |  | **OR (95% CI)** | ***p*-value** |
| **Upward temperature shifts** | | |  |  |  |  |  |  |  |
|  | 0 | **1.009 (1.005,1.013)** | **<0.001** |  | **1.014 (1.006,1.022)** | **<0.001** |  | **1.007 (1.001,1.012)** | **0.014** |
|  | 1 | 1.002 (0.999,1.006) | 0.189 |  | 0.999 (0.993,1.005) | 0.742 |  | 1.004 (1.000,1.008) | 0.069 |
|  | 2 | 1.001 (0.999,1.004) | 0.196 |  | 1.000 (0.996,1.004) | 0.892 |  | 1.002 (1.000,1.005) | 0.079 |
|  | 3 | 1.002 (1.000,1.004) | 0.062 |  | 1.004 (1.000,1.007) | 0.077 |  | 1.002 (0.999,1.004) | 0.229 |
|  | 4 | 1.002 (0.999,1.004) | 0.161 |  | 1.003 (1.000,1.007) | 0.077 |  | 1.001 (0.998,1.003) | 0.527 |
|  | 5 | 1.000 (0.999,1.002) | 0.878 |  | 1.001 (0.998,1.004) | 0.683 |  | 1.000 (0.998,1.002) | 0.987 |
|  | 6 | 0.998 (0.995,1.002) | 0.318 |  | 0.996 (0.991,1.002) | 0.234 |  | 0.999 (0.995,1.003) | 0.690 |
| **Downward temperature shifts** | | |  |  |  |  |  |  |  |
|  | 0 | 0.994 (0.990,0.998) | 0.007 |  | 0.994 (0.987,1.001) | 0.103 |  | 0.995 (0.990,0.999) | 0.029 |
|  | 1 | 1.001 (0.998,1.004) | 0.552 |  | 1.004 (0.998,1.009) | 0.162 |  | 1.000 (0.996,1.003) | 0.806 |
|  | 2 | **1.003 (1.001,1.005)** | **0.014** |  | **1.006 (1.002,1.010)** | **0.001** |  | 1.001 (0.998,1.004) | 0.438 |
|  | 3 | 1.002 (1.000,1.004) | 0.064 |  | **1.005 (1.001,1.008)** | **0.011** |  | 1.001 (0.998,1.003) | 0.560 |
|  | 4 | 1.001 (0.999,1.003) | 0.420 |  | 1.003 (0.999,1.006) | 0.146 |  | 1.000 (0.998,1.003) | 0.939 |
|  | 5 | 0.999 (0.998,1.001) | 0.452 |  | 1.000 (0.997,1.003) | 0.994 |  | 0.999 (0.997,1.001) | 0.406 |
|  | 6 | 0.998 (0.995,1.001) | 0.171 |  | 0.997 (0.992,1.003) | 0.315 |  | 0.998 (0.994,1.002) | 0.342 |

Note: MI, myocardial infarction. STEMI, ST-segment elevation myocardial infarction. NSTEMI, non-ST-segment elevation myocardial infarction. Total MI refers to all types of MI hospitalizations combined. OR, odds ratio. CI, confidence interval.
