# Supplementary material for: Short-term exposure to ambient temperature variability and myocardial infarction hospital admissions: A nationwide case-crossover study in Sweden
Source: PLoS Med. 2025 May 20;22(5):e1004607. doi: 10.1371/journal.pmed.1004607 (PMC12091774; doi:10.1371/journal.pmed.1004607)
Supplement: S3 Table — Note: MI, myocardial infarction; OR, odds ratio; CI, confidence interval. (DOCX) [file pmed.1004607.s006.docx]

### **Table S3. Associations of short-term exposures to temperature variability (upward and downward temperature shifts) with first and recurrent MI hospital admissions.**

| **Temperature variability** | **Lag** | **First MI** | |  | **Recurrent MI** | |
| --- | --- | --- | --- | --- | --- | --- |
|  |  | **OR (95% CI)** | ***p*-value** |  | **OR (95% CI)** | ***p*-value** |
| **Upward temperature shifts** | 0 | 1.009 (1.004,1.014) | <0.001 |  | 1.013 (1.006,1.020) | <0.001 |
| **Downward temperature shifts** | 2 | 1.003 (0.998,1.007) | 0.204 |  | 1.001 (0.995,1.008) | 0.721 |

Note: MI, myocardial infarction. OR, odds ratio. CI, confidence interval.
