# Supplementary material for: Short-term exposure to ambient temperature variability and myocardial infarction hospital admissions: A nationwide case-crossover study in Sweden
Source: PLoS Med. 2025 May 20;22(5):e1004607. doi: 10.1371/journal.pmed.1004607 (PMC12091774; doi:10.1371/journal.pmed.1004607)
Supplement: S4 Table — Note: MI, myocardial infarction; STEMI, ST-segment elevation myocardial infarction; NSTEMI, non-ST-segment elevation myocardial infarction. Total MI refers to all types of MI hospitalizations combined. OR, odds ratio; CI, confidence interval. (DOCX) [file pmed.1004607.s007.docx]

### **Table S4. Associations of upward temperature shifts at lag 0 day and downward temperature shifts at lag 2 day with total MI, STEMI, and NSTEMI hospitalizations in the central, southern, and northern regions of Sweden.**

| **Temperature variability** | **Region** | **Total MI** | |  | **STEMI** | |  | **NSTEMI** | |
| --- | --- | --- | --- | --- | --- | --- | --- | --- | --- |
|  |  | **OR**  **(95% CI)** | ***p*-value** |  | **OR**  **(95% CI)** | ***p*-value** |  | **OR**  **(95% CI)** | ***p*-value** |
| **Upward temperature shifts** | | |  |  |  |  |  |  |  |
|  | North | 1.012 (1.003,1.020) | 0.005 |  | 1.013 (0.998,1.028) | 0.079 |  | 1.011 (1.001,1.021) | 0.027 |
|  | Center | 1.011 (1.005,1.017) | <0.001 |  | 1.013 (1.002,1.024) | 0.023 |  | 1.011 (1.004,1.018) | 0.003 |
|  | South | 1.009 (1.003,1.015) | 0.005 |  | 1.015 (1.004,1.026) | 0.008 |  | 1.006 (0.999,1.014) | 0.110 |
| **Downward temperature shifts** | | |  |  |  |  |  |  |  |
|  | North | 0.999 (0.991,1.007) | 0.843 |  | 1.004 (0.990,1.018) | 0.579 |  | 0.997 (0.988,1.007) | 0.596 |
|  | Center | 1.004 (0.998,1.010) | 0.155 |  | 1.011 (1.001,1.022) | 0.035 |  | 1.001 (0.994,1.008) | 0.811 |
|  | South | 1.002 (0.996,1.008) | 0.455 |  | 1.009 (0.999,1.020) | 0.086 |  | 0.999 (0.992,1.007) | 0.867 |

Note: MI, myocardial infarction. STEMI, ST-segment elevation myocardial infarction. NSTEMI, non-ST-segment elevation myocardial infarction. Total MI refers to all types of MI hospitalizations combined. OR, odds ratio. CI, confidence interval.
