# Supplementary figures and images for: Short-term exposure to ambient temperature variability and myocardial infarction hospital admissions: A nationwide case-crossover study in Sweden
Source: PLoS Med. 2025 May 20;22(5):e1004607. doi: 10.1371/journal.pmed.1004607 (PMC12091774; doi:10.1371/journal.pmed.1004607)

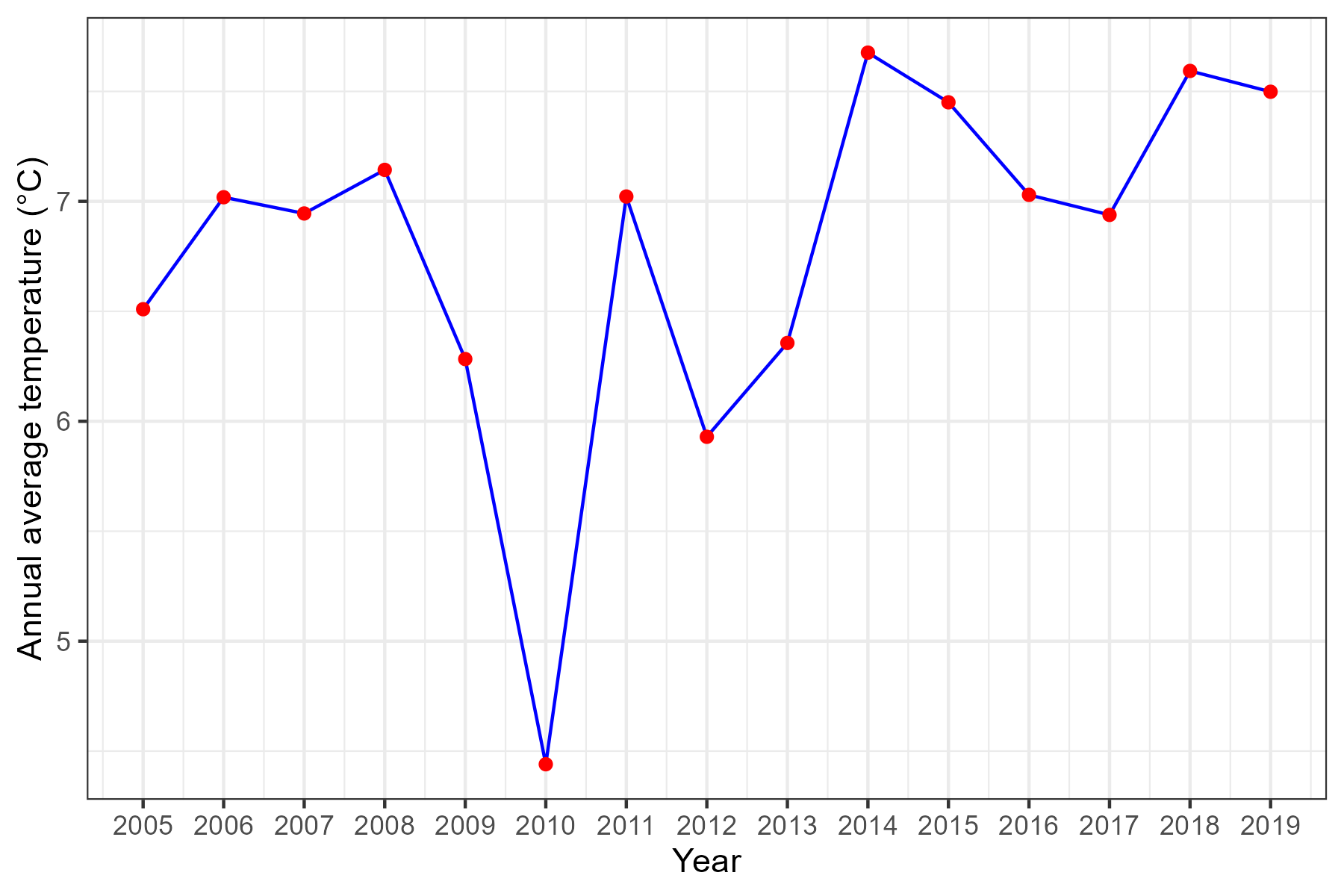


### **Figure S1. Yearly average of mean temperature trends in Sweden (2005-2019)**

Supplement: S1 Fig — (DOCX) [file pmed.1004607.s008.docx]
