# Supplementary material for: Short-term exposure to ambient temperature variability and myocardial infarction hospital admissions: A nationwide case-crossover study in Sweden
Source: PLoS Med. 2025 May 20;22(5):e1004607. doi: 10.1371/journal.pmed.1004607 (PMC12091774; doi:10.1371/journal.pmed.1004607)
Supplement: S3 Fig — Note: LR-test, likelihood ratio test (temperature variability was included as natural cubic spline with 3dfs versus temperature variability was included as linear). MI, myocardial infarction; STEMI, ST-segment elevation myocardial infarction; NSTEMI, non-ST-segment elevation myocardial infarction. Total MI refers to all types of MI hospitalizations combined. (DOCX) [file pmed.1004607.s010.docx]

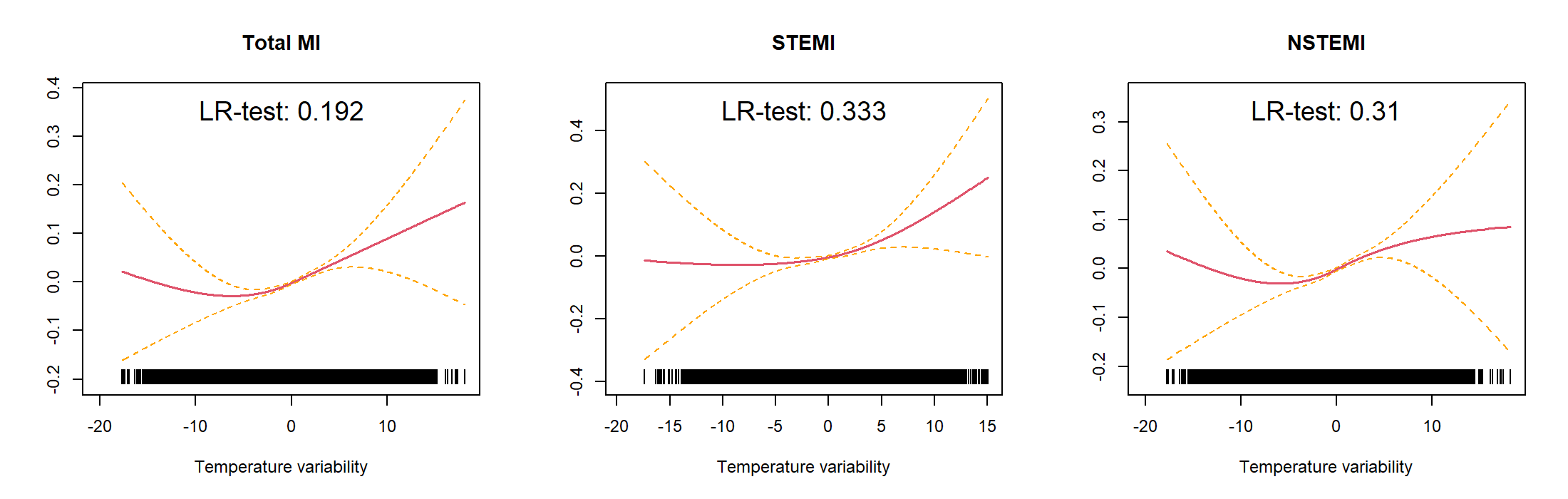


### **Figure S3. Exposure-response functions of temperature variability and total MI, STEMI, and NSTEMI hospital admissions**

Note: LR-test, likelihood ratio test (temperature variability was included as natural cubic spline with 3dfs vs. temperature variability was included as linear). MI, myocardial infarction. STEMI, ST-segment elevation myocardial infarction. NSTEMI, non-ST-segment elevation myocardial infarction. Total MI refers to all types of MI hospitalizations combined.
