# Supplementary material for: Short-term exposure to ambient temperature variability and myocardial infarction hospital admissions: A nationwide case-crossover study in Sweden
Source: PLoS Med. 2025 May 20;22(5):e1004607. doi: 10.1371/journal.pmed.1004607 (PMC12091774; doi:10.1371/journal.pmed.1004607)
Supplement: S4 Fig — Note: PM2.5, particulate matter with diameter ≤2.5 micrometers. NO2, nitrogen dioxide; O3, ozone. (DOCX) [file pmed.1004607.s011.docx]

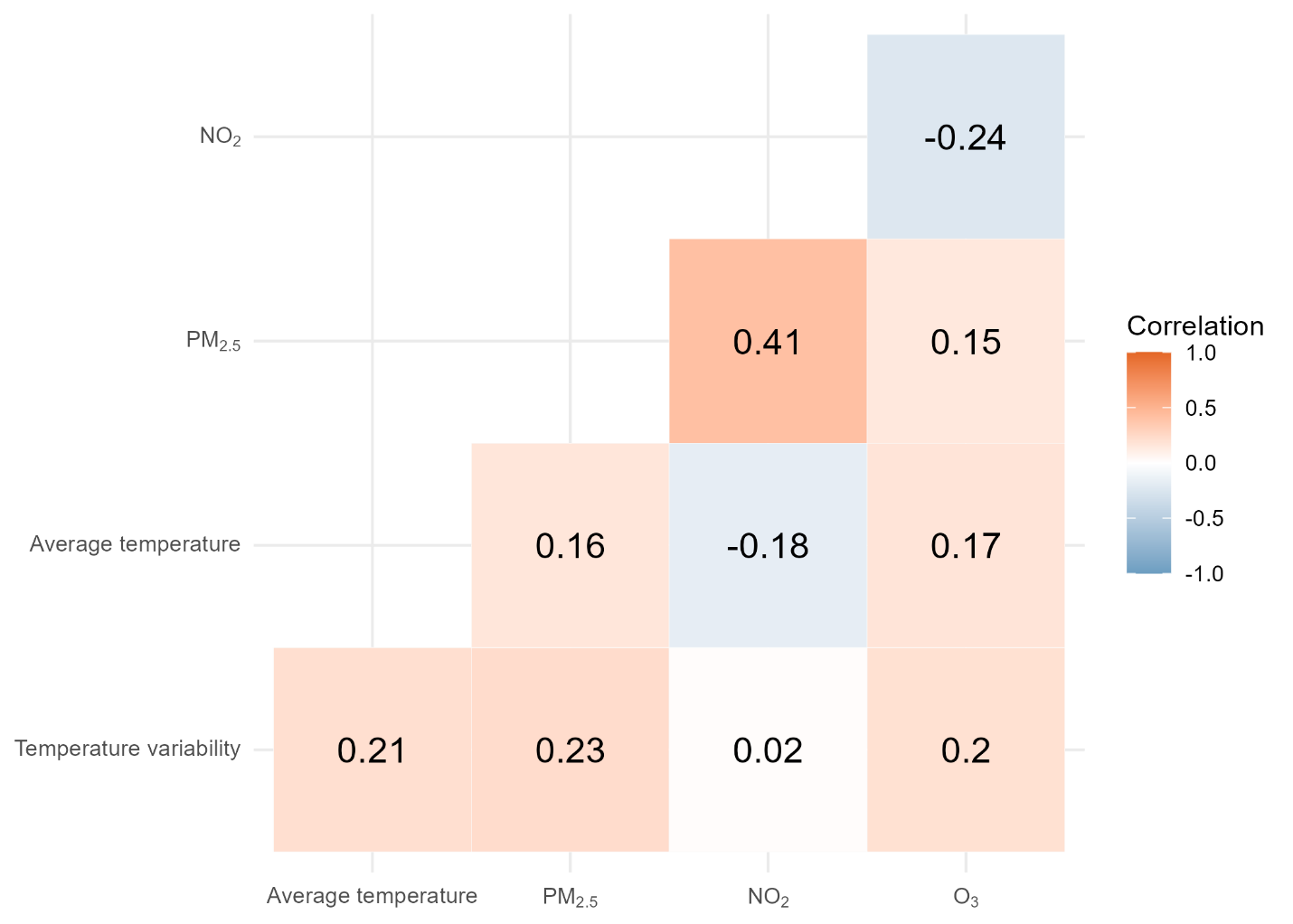


### **Figure S4. Spearman correlations between ambient temperatures and ambient pollutants variables.**

Note: PM_2.5,_ particulate matter with diameter ≤2.5 micrometers_._ NO_2,_ nitrogen dioxide. O_3,_ ozone.
