# Supplementary material for: Short-term exposure to ambient temperature variability and myocardial infarction hospital admissions: A nationwide case-crossover study in Sweden
Source: PLoS Med. 2025 May 20;22(5):e1004607. doi: 10.1371/journal.pmed.1004607 (PMC12091774; doi:10.1371/journal.pmed.1004607)
Supplement: S5 Fig — Note: MI, myocardial infarction; STEMI, ST-segment elevation myocardial infarction; NSTEMI, non-ST-segment elevation myocardial infarction. Total MI refers to all types of MI hospitalizations combined. OR, odds ratio; CI, confidence interval. (DOCX) [file pmed.1004607.s012.docx]

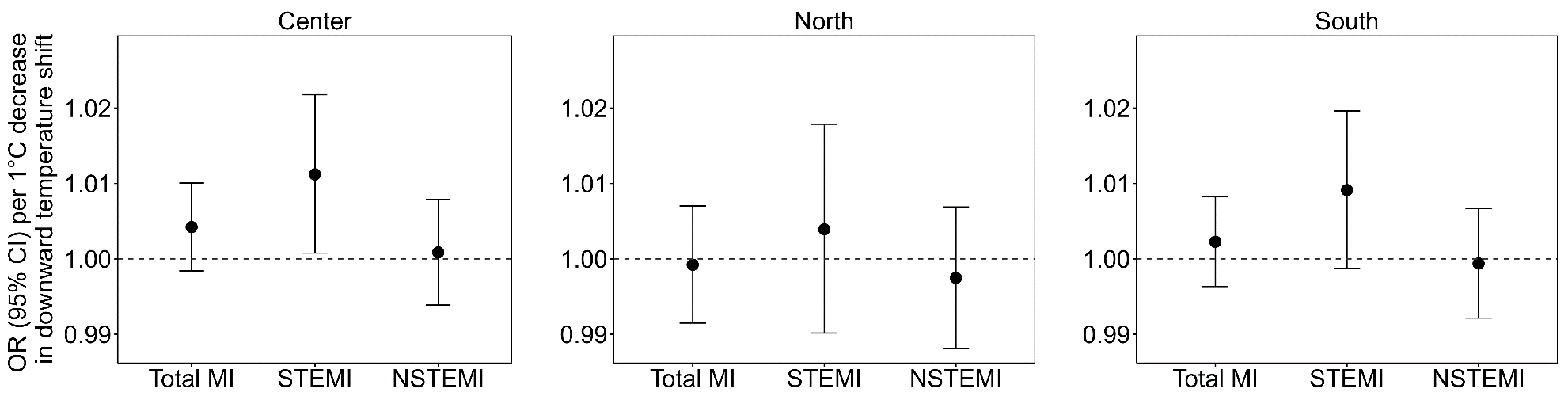


### **Figure S5. Associations of downward temperature shifts (temperature variability) at lag 2 day with total MI, STEMI, and NSTEMI hospitalizations in the central, southern, and northern regions of Sweden.**

Note: MI, myocardial infarction. STEMI, ST-segment elevation myocardial infarction. NSTEMI, non-ST-segment elevation myocardial infarction. Total MI refers to all types of MI hospitalizations combined. OR, odds ratio. CI, confidence interval.
