# Supplementary material for: Short-term exposure to ambient temperature variability and myocardial infarction hospital admissions: A nationwide case-crossover study in Sweden
Source: PLoS Med. 2025 May 20;22(5):e1004607. doi: 10.1371/journal.pmed.1004607 (PMC12091774; doi:10.1371/journal.pmed.1004607)
Supplement: S7 Fig — Note: MI, myocardial infarction; STEMI, ST-segment elevation myocardial infarction; NSTEMI, non-ST-segment elevation myocardial infarction. Total MI refers to all types of MI hospitalizations combined. ACE inhibitors, angiotensin-converting enzyme inhibitors. A2 blockers, angiotensin II receptor blockers. DOAC, including apixaban, dabigatran etexilate, edoxaban, and rivaroxaban. OR, odds ratio; CI, confidence interval. (DOCX) [file pmed.1004607.s014.docx]

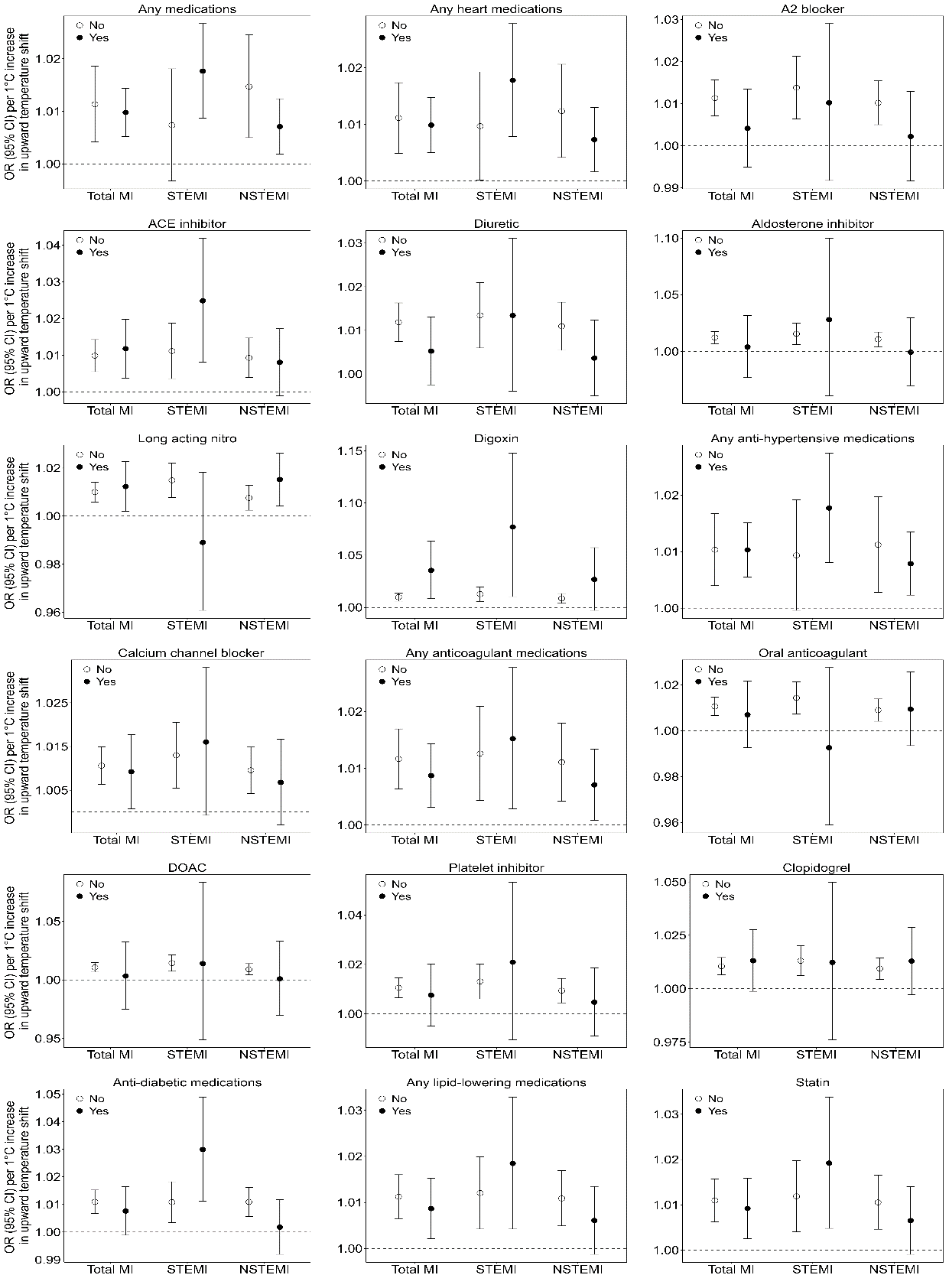


### **Figure S7. Effect modifications of upward temperature shift on MI hospital admissions by history of medications at lag 0 day.**

Note: MI, myocardial infarction. STEMI, ST-segment elevation myocardial infarction. NSTEMI, non-ST-segment elevation myocardial infarction. Total MI refers to all types of MI hospitalizations combined. ACE inhibitors, angiotensin-converting enzyme inhibitors. A2 blockers, angiotensin II receptor blockers. DOAC, including apixaban, dabigatran etexilate, edoxaban, and rivaroxaban. OR, odds ratio. CI, confidence interval.
