# Supplementary material for: Short-term exposure to ambient temperature variability and myocardial infarction hospital admissions: A nationwide case-crossover study in Sweden
Source: PLoS Med. 2025 May 20;22(5):e1004607. doi: 10.1371/journal.pmed.1004607 (PMC12091774; doi:10.1371/journal.pmed.1004607)
Supplement: S8 Fig — Note: MI, myocardial infarction; STEMI, ST-segment elevation myocardial infarction; NSTEMI, non-ST-segment elevation myocardial infarction. Total MI refers to all types of MI hospitalizations combined. PM2.5, particulate matter with diameter ≤2.5 micrometers. NO2, nitrogen dioxide; O3, ozone; OR, odds ratio; CI, confidence interval. (DOCX) [file pmed.1004607.s015.docx]

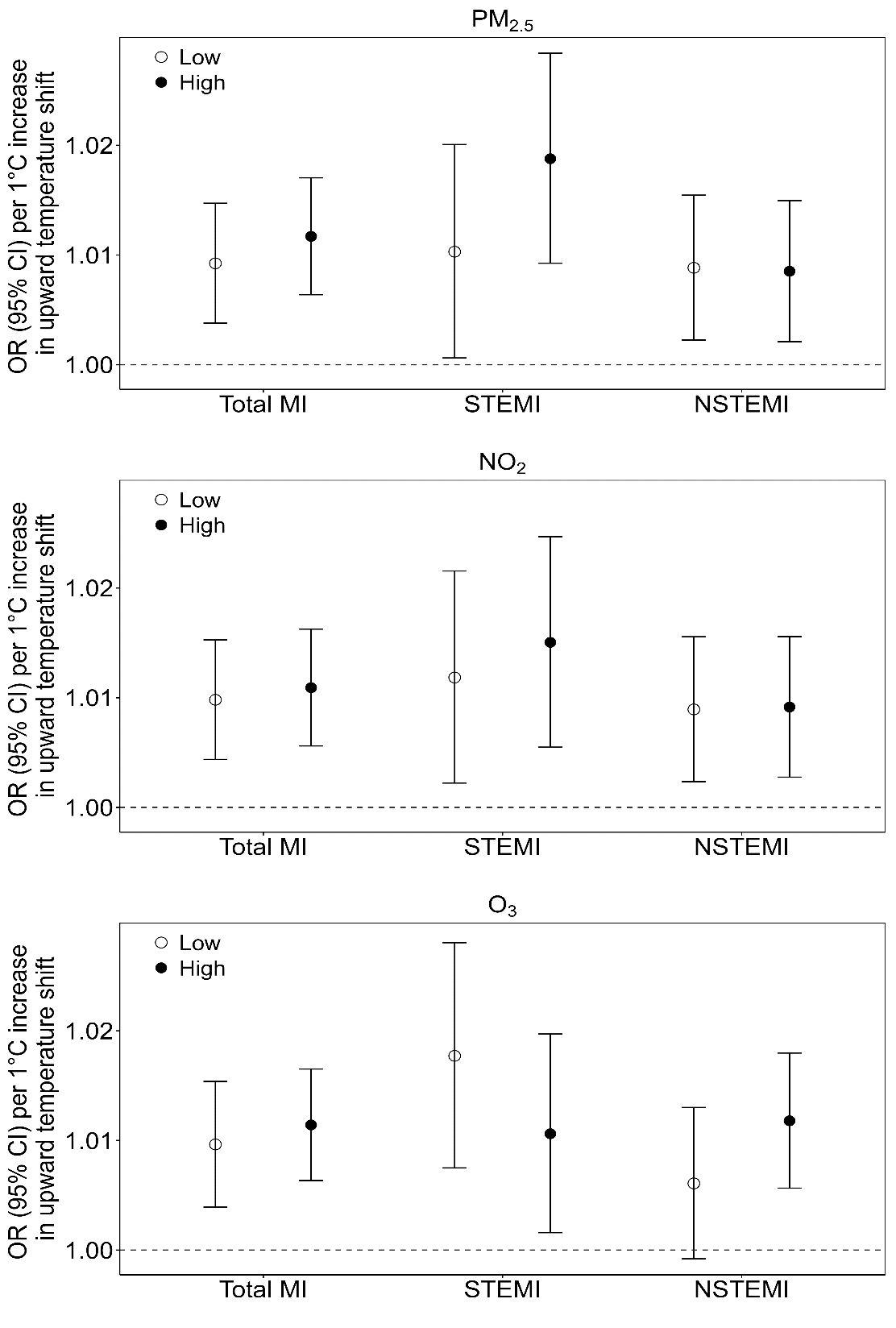


### **Figure S8. Effect modifications of upward temperature shift on MI hospital admissions by air pollutants at lag 0 day.**

Note: MI, myocardial infarction. STEMI, ST-segment elevation myocardial infarction. NSTEMI, non-ST-segment elevation myocardial infarction. Total MI refers to all types of MI hospitalizations combined. PM_2.5,_ particulate matter with diameter ≤2.5 micrometers_._ NO_2,_ nitrogen dioxide. O_3,_ ozone. OR, odds ratio. CI, confidence interval.
