# Supplementary material for: Short-term exposure to ambient temperature variability and myocardial infarction hospital admissions: A nationwide case-crossover study in Sweden
Source: PLoS Med. 2025 May 20;22(5):e1004607. doi: 10.1371/journal.pmed.1004607 (PMC12091774; doi:10.1371/journal.pmed.1004607)
Supplement: S11 Fig — Note: Red error bars represent p-value for the interaction term <0.05. MI, myocardial infarction; STEMI, ST-segment elevation myocardial infarction; NSTEMI, non-ST-segment elevation myocardial infarction. Total MI refers to all types of MI hospitalizations combined. ACE inhibitors, angiotensin-converting enzyme inhibitors. A2 blockers, angiotensin II receptor blockers. DOAC, including apixaban, dabigatran etexilate, edoxaban, and rivaroxaban. OR, odds ratio; CI, confidence interval. (DOCX) [file pmed.1004607.s018.docx]

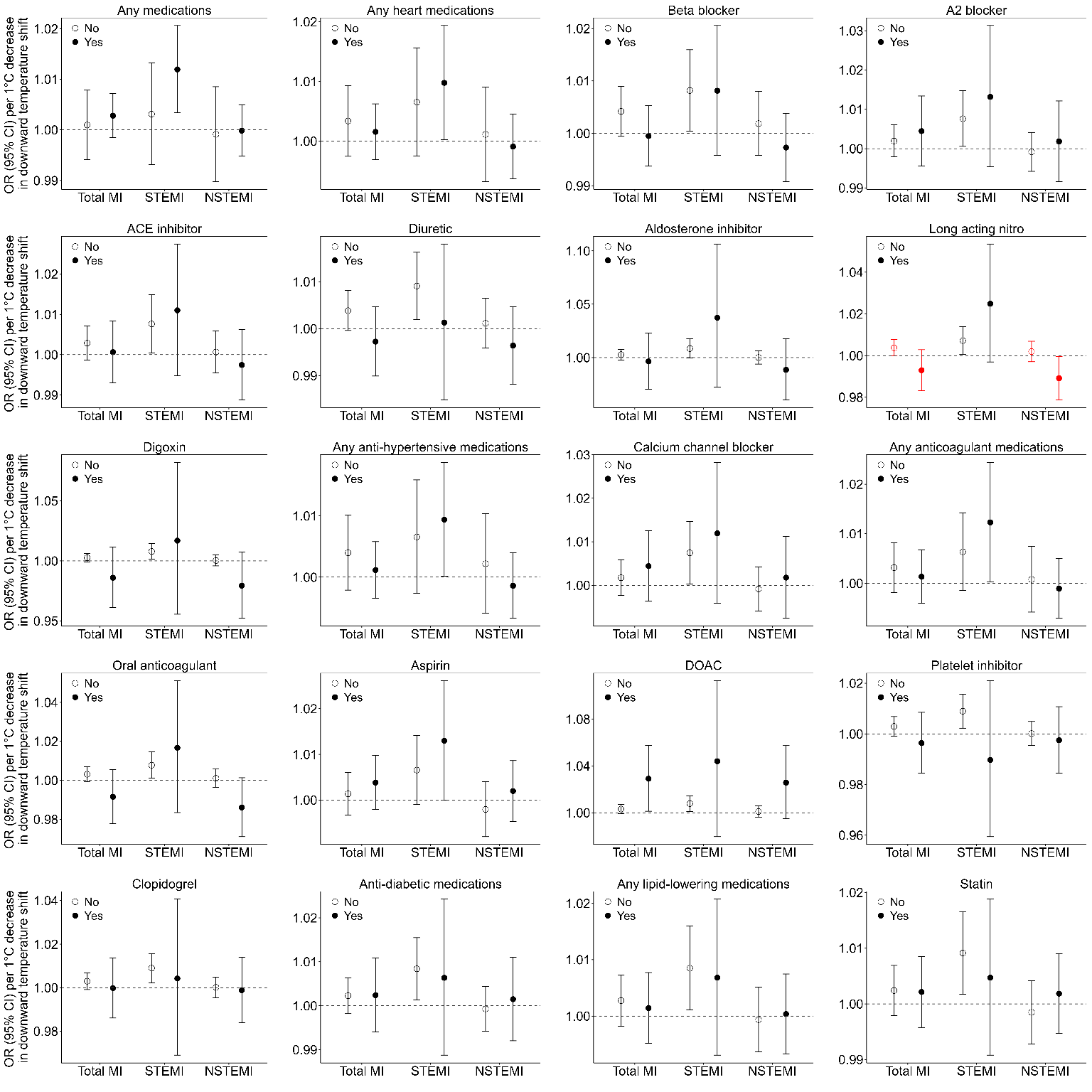


### **Figure S11. Effect modifications of downward temperature shift on MI hospital admissions by history of medications at lag 2 day.**

Note: Red error bars represent *p*-value for the interaction term < 0.05. MI, myocardial infarction. STEMI, ST-segment elevation myocardial infarction. NSTEMI, non-ST-segment elevation myocardial infarction. Total MI refers to all types of MI hospitalizations combined. ACE inhibitors, angiotensin-converting enzyme inhibitors. A2 blockers, angiotensin II receptor blockers. DOAC, including apixaban, dabigatran etexilate, edoxaban, and rivaroxaban. OR, odds ratio. CI, confidence interval.
